# The gastrin and cholecystokinin receptors mediated signaling network: a scaffold for data analysis and new hypotheses on regulatory mechanisms

**DOI:** 10.1186/s12918-015-0181-z

**Published:** 2015-07-24

**Authors:** Sushil Tripathi, Åsmund Flobak, Konika Chawla, Anaïs Baudot, Torunn Bruland, Liv Thommesen, Martin Kuiper, Astrid Lægreid

**Affiliations:** Department of Cancer Research and Molecular Medicine, Norwegian University of Science and Technology (NTNU), N-7489 Trondheim, Norway; Department of Biology, Norwegian University of Science and Technology (NTNU), N-7491 Trondheim, Norway; I2M, Marseilles Institute of Mathematics CNRS - AMU, Case 907, 13288 Marseille, Cedex 9 France; Department of Technology, Sør-Trøndelag University College, N-7004 Trondheim, Norway; Institute of Cancer Research and Molecular Medicine, Norwegian University of Science and Technology (NTNU), N-7489 Trondheim, Norway

**Keywords:** Cholecystokinin receptor, Map, Model, Modules, Network, Protein-protein interaction, Signaling pathway, Gastrin

## Abstract

**Background:**

The gastrointestinal peptide hormones cholecystokinin and gastrin exert their biological functions via cholecystokinin receptors CCK1R and CCK2R respectively. Gastrin, a central regulator of gastric acid secretion, is involved in growth and differentiation of gastric and colonic mucosa, and there is evidence that it is pro-carcinogenic. Cholecystokinin is implicated in digestion, appetite control and body weight regulation, and may play a role in several digestive disorders.

**Results:**

We performed a detailed analysis of the literature reporting experimental evidence on signaling pathways triggered by CCK1R and CCK2R, in order to create a comprehensive map of gastrin and cholecystokinin-mediated intracellular signaling cascades. The resulting signaling map captures 413 reactions involving 530 molecular species, and incorporates the currently available knowledge into one integrated signaling network. The decomposition of the signaling map into sub-networks revealed 18 modules that represent higher-level structures of the signaling map. These modules allow a more compact mapping of intracellular signaling reactions to known cell behavioral outcomes such as proliferation, migration and apoptosis. The integration of large-scale protein-protein interaction data to this literature-based signaling map in combination with topological analyses allowed us to identify 70 proteins able to increase the compactness of the map. These proteins represent experimentally testable hypotheses for gaining new knowledge on gastrin- and cholecystokinin receptor signaling. The CCKR map is freely available both in a downloadable, machine-readable SBML-compatible format and as a web resource through PAYAO (http://sblab.celldesigner.org:18080/Payao11/bin/).

**Conclusion:**

We have demonstrated how a literature-based CCKR signaling map together with its protein interaction extensions can be analyzed to generate new hypotheses on molecular mechanisms involved in gastrin- and cholecystokinin-mediated regulation of cellular processes.

**Electronic supplementary material:**

The online version of this article (doi:10.1186/s12918-015-0181-z) contains supplementary material, which is available to authorized users.

## Background

Gastrin and cholecystokinin (CCK) are gastrointestinal peptide hormones that share a common C-terminal pentapeptide amide [1]. Gastrin, produced in G-cells of the gastric antrum, is the central regulator of gastric acid secretion but also regulates growth and differentiation of gastric and colonic mucosa [2]. CCK, produced primarily in I-cells of the small intestine, is involved in physiological processes such as digestion, appetite control and body weight regulation [3]. The scientific interest in these hormones is further strengthened by their roles in several diseases. Indeed, CCK has been implicated in acute pancreatitis [4–6], obesity [7, 8], irritable bowel syndrome [9] and gall bladder disease [10, 11]. Gastrin is known to be pro-carcinogenic, affecting proliferation, angiogenesis and apoptosis [2], and is a risk co-factor for gastric carcinogenesis and atrophy upon *Helicobacter pylori* infection [12, 13]. In order to efficiently study and understand the molecular mechanisms triggered by gastrin and cholecystokinin, detailed knowledge concerning the signaling pathways they regulate is paramount.

Information concerning intracellular signaling is commonly retrieved from databases such as Reactome [14] and KEGG [15]. However, none of these resources currently specify which specific molecular events are known to take place in response to gastrin or CCK. Researchers in need of such knowledge therefore must spend significant time reviewing current literature in order to gain an exhaustive and up-to-date understanding of the signaling network. A comprehensive map of gastrin and CCK intracellular signaling pathways would significantly assist in the study of normal or aberrant cholecystokinin receptor (CCKR) signaling.

In the past decade, several manually constructed maps of signaling events have been published [16–25], each providing solid foundations for a systems understanding of the signaling mechanisms. The present work extends this approach to the domain of CCKR signaling by providing a comprehensive literature-based CCKR signaling map that comprises 530 molecular species and 431 reactions, considerably extending previously compiled knowledge on CCK2R signaling [10, 26] including CCK1R downstream events.

Partitioning the total CCKR signaling map into sub-networks using the BiNoM tool [27] resulted in 18 modules that coordinate with each other to elicit the diverse intracellular signaling responses to gastrin and/or CCK. Finally, we used the CCKR map as a scaffold for protein-protein interaction (PPI) data integration assisted by PathExpand [28], in order to predict novel components of the signaling network. This resulted in the identification of 70 new proteins tightly connected to the CCKR signaling map, making them prime candidates for future experimental work aimed at further extending knowledge on regulation of CCKR mediated signaling mechanisms.

## Methods

### Construction of the CCKR map from literature

**Fig. 1:**
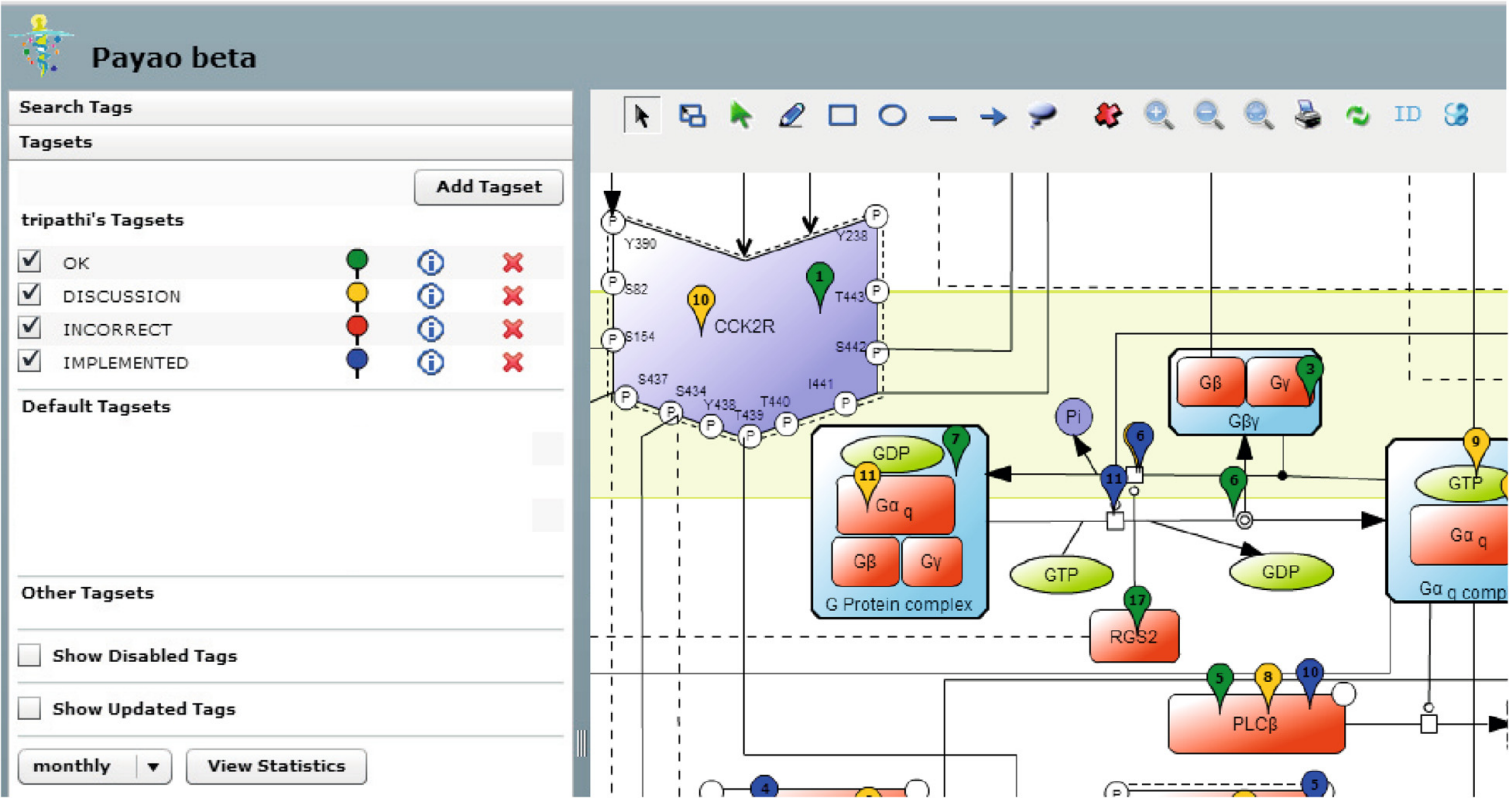
CCKR map collaborative curation in Payao. Shows detail from CCKR map in Payao web tool illustrating collaborative efforts involving five CCKR map curators. Evaluation- and action points for reactions, components and cellular localizations are indicated with the Tagsets ‘OK’ (*green*), ‘DISCUSSION’ (*yellow*), ‘INCORRECT’ (*red*) and ‘IMPLEMENTED’ (*blue*)

The CCKR map was constructed using CellDesigner 4.2, a structured diagram editor for drawing gene-regulatory and biochemical networks, following the Systems Biology Graphical Notation (SBGN) standard for process diagrams [29] and the Systems Biology Mark-up Language (SBML) for model representation [30]. The MIRIAM (Minimum Information Requested In the Annotation of Models) guidelines were followed to characterize each species in the map [31].

i). Knowledge encoded in the CCKR map was obtained from scientific publications that were identified by searching for different combinations of cholecystokinin (CCK)/CCK1R and gastrin (G-17)/CCK2R in PubMed or through various literature mining tools, e.g. LitInspector (http://www.genomatix.de/solutions/genomatix-software-suite.html) [32] and iHOP (http://www.ihop-net.org/UniPub/iHOP/) [33]. We adopted two main criteria as guidelines for including a scientific paper for the information extraction, namely that it must contain:
  1. evidence that the reported signaling event is mediated by the specific interaction of CCK or gastrin (G-17) with their receptors CCK1R and CCK2R.
  2. signaling information to allow for linkage of a new CCKR map component to its upstream and/or downstream regulators in CCKR signaling.
ii). CellDesigner species and reaction “note” features were used to record PubMed IDs (PMID), cell-type specific information for each reaction, and the interacting components in the CCKR map.
iii). Final curation and quality control was done in a collaborative effort involving five different research group members who collectively used the community curation platform PAYAO (http://www.payaologue.org) [34], enabling efficient exchange of comments and tags. Consensus and critical comments from each annotator about the precise representation of reactions, components, and their cellular localization were discussed and implemented (Fig. 1). Finally, the CCKR map was published with an open source license for the whole scientific community through PAYAO [34] (map available at: http://sblab.celldesigner.org:18080/Payao11/bin/). The CCKR map is also available in the SBML data exchange format (Additional file 1), and we are also in the process of submitting this map to the PANTHER database [35].

We welcome comments and tags from a community of curators to continue increasing the quality of this CCKR signaling map and will keep it up to date with our growing biological understanding.

### Analysis of the CCKR map

Network topology studies were performed by importing the CellDesigner-generated SBML file of the CCKR map to Cytoscape version 2.8 [36] using the BiNoM plugin [27], after removing connections downstream of the transcription factor layer. BiNoM considers both ‘reaction’ and ‘species’ of a CellDesigner map as a node. The Cytoscape version of the CCKR map consisted of 807 nodes (475 species and 332 reactions) with 963 edges. Node degree and closeness centrality were calculated using the ‘Network Analysis’ plugin [37] in Cytoscape, assuming the network to be undirected. The number of nodes connected directly to a particular node defines its node degree *k*, and the node degrees of all nodes in the network represent the ‘degree distribution’ of the network. Nodes with degree *k* > 5 were termed ‘hubs’.

### BiNoM decomposition of CCKR map into modules

We used the ‘prune the graph’ function of BiNoM to automatically separate the strongly connected component (SCC), i.e. the central cyclic motif of the map, from more loosely connected upstream and downstream species. The SCC was further decomposed into smallest sub-networks with the function ‘extract material components’. Next, some manual curation work was performed: sub-networks with 50 % or more overlapping nodes were clustered together while the large sub-networks were decomposed further until each sub-network contained a unique central cyclic motif. Any unconnected nodes were removed from the sub-networks, yielding a total of 18 sub-networks or modules. Next, these 18 modules were merged together and the resulting network was compared with the initial map to check for completeness in terms of any missing interactions or nodes. The complete map and its 18 decomposed modules are available as a Cytoscape session file (Additional file 2).

### Protein-protein interaction based expansion of the CCKR map

PPI data were downloaded using PSICQUIC (all databases, version June 2012), and filtered for binary physical interactions based on PSI-MI controlled vocabulary experimental method descriptions, following the procedure in [38] (Charles E. Chapple, personal communication). We then tested each of the 4119 proteins found to interact with CCKR signaling map proteins with the PathExpand method [28] to seek for tightly linked protein interactors that enhance the network compactness of the CCKR map by leading to higher node degree, betweenness and average local clustering as well as decreasing the shortest path lengths (for details see [28]) .

## Results

CCK and gastrin impinge on cellular functioning by binding to two different G protein-coupled receptors, CCK1R and CCK2R, respectively, located at the surface of multiple cell types in peripheral organs such as the gastrointestinal tract, the pancreas, and the gall bladder [39]. Today, no comprehensive resource exists that compiles current knowledge on CCKR activated signaling pathways. Gastrin has a strong preference for CCK2R, while CCK can activate both receptors with similar affinities [10]. Most cell types responsive to one or both peptide hormones express only one CCK-receptor variant. However, a range of normal and cancer cells in whole organisms as well as model cell lines (for instance the rat pancreatic acinar cell derived cell-line AR42J [40]) express both CCK1R and CCK2R.

### The CCKR signaling map

**Fig. 2:**
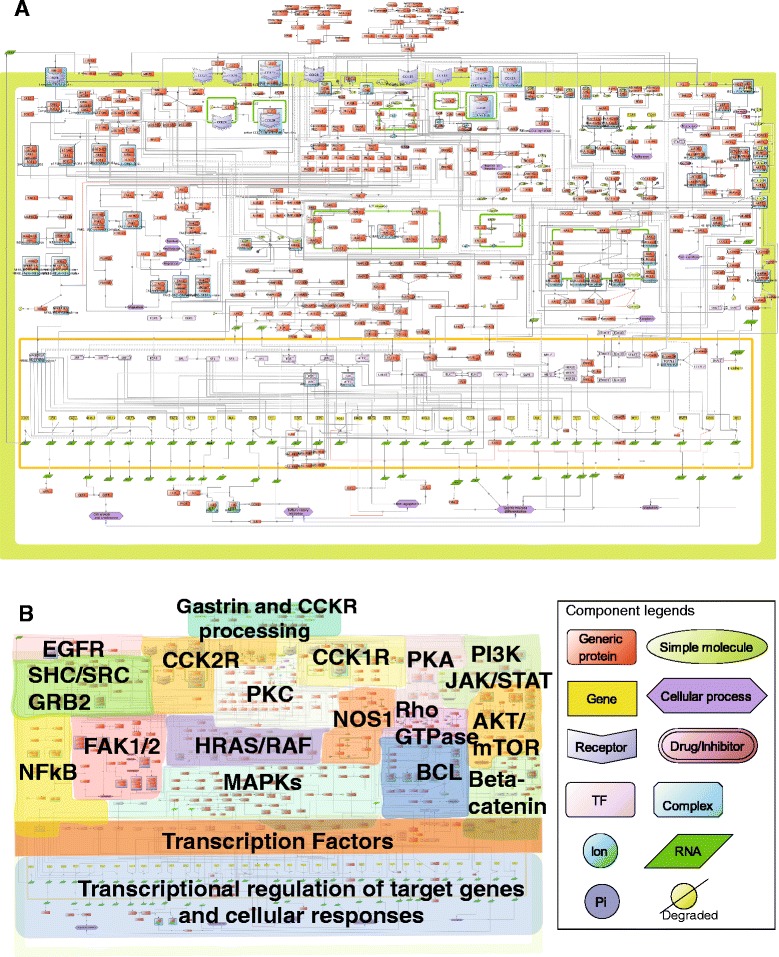
CCKR map (CellDesigner^TM^ 4.2). **a** Literature curated comprehensive map of the CCK1R, CCK2R mediated signaling pathways comprising 530 species and 431 reactions (see Table 1 for further details). The graphical representation is also available as an SBML file (Additional file 1) **b** Navigation map to track components and signaling cascades in the detailed map shown in part A

We present a CCKR signaling map built with biological inferences from more than 250 scientific publications (including original articles and reviews), and based on experiments performed in 37 different cell lines representing a wide array of cell types (Fig. 2, Methods).

The CCKR map encompasses 199 unique proteins and their relationships to complexes, genes, and small molecules (including some inhibitors, e.g. rapamycin, wortmannin), describing a total of 530 molecular species and 431 reactions experimentally documented to play a role in CCKR signaling. The reactions include molecular state transitions (e.g. activation, phosphorylation), transport, heterodimer associations/dissociations as well as transcriptional regulation of target genes by transcription factors, which are reported to be associated with the regulation of gastrointestinal hormone responsive biological processes (Table 1).

**Table 1:** Overview of the CCKR map

*Species*		*Reactions*	
Category	Number (530)	Category	Number (431)
Proteins	314 (199 unique)	Heterodimer associations and dissociations	45
Complexes	63	State transitions	193
Genes	36	Transports	60
RNAs	63 (35 unique)	Transcriptions and translations	41
Other^a^	54	Other^b^	92

^a^Simple molecules, phenotype, degraded products, ions, drugs, unknown molecules

^b^Known transition omitted, truncation, unknown transitions, unknown negative influence, positive influence

Figure 2a displays a detailed view of the map depicting the two ligands gastrin and CCK, their biogenesis and processing, their binding to the two G protein coupled receptors CCK1R and CCK2R and the ensuing signal transduction pathways including activation of transcription factors and downstream target genes reported to be triggered in response to gastrin and/or CCK. Moreover, a number of cellular processes influenced by specific CCKR signaling events are indicated, including proliferation, migration, differentiation, anti-apoptosis and inflammation. Figure 2b is an aid for the reader to navigate in Fig. 2a, as it indicates the position of major pathways involved, including protein kinase C (PKC)-dependent activation of MAPK cascades, PI3K-mTOR signaling, protein kinase A (PKA)-dependent pathway, β-catenin, and Rho-GTPase-Bcl cascades.

**Fig. 3:**
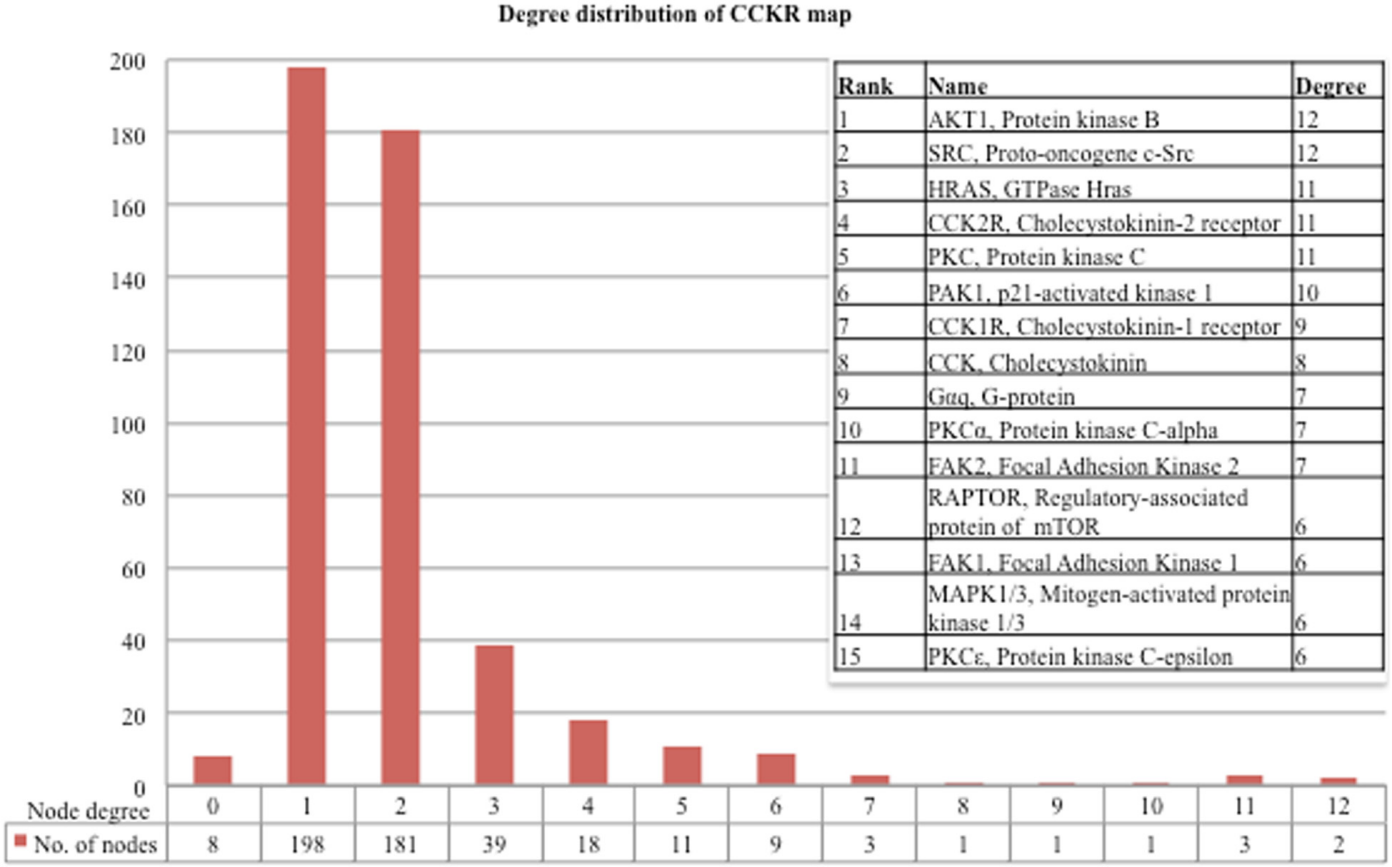
Degree distribution of the CCKR map proteins

Network topology analysis of the CCKR map indicated scale-free characteristics, with the majority of the proteins connected to only few other network proteins (Fig. 3). The 6 most highly connected proteins include four protein kinases AKT1, SRC, PKC and PAK1, and the small GTPase HRAS (Fig. 3, inserted table).

### Signaling pathways shared by CCK1R and CCK2R

Downstream signaling mechanisms shared by CCK1R and CCK2R include the trimeric guanine nucleotide binding alpha q protein (G-protein α_q_), PKC dependent phosphorylation of adaptor protein SHC - transforming protein C, and its association with GRB2/SOS1 leading to activation of the HRAS/RAF/MAPK1/3 cascade [10, 41, 42]. Other shared pathways involve MAP3K11-mediated regulation of MAPK8, −9 and −10 and p38MAPK (MAPK14), PRKD1 (in PKC signaling), PI3K, AKT1, FAK1, JAK2-STAT3 and Rho GTPase [10, 41–44]. Both receptors activate PKC isoforms PKCα, −δ, −ε, −θ and −ζ [10, 45–47]. Transcription factors reported downstream of both CCK1R and CCK2R receptors include NFκB, CREB1, ELK1 and AP1.

### CCK1R-specific signaling pathways

Two trimeric G-proteins appear to be regulated only by CCK1R. One is G alpha S (Gα_s_) [48], which leads to Protein kinase A activation via adenylate cyclase catalyzed cAMP production, and the other is G alpha 13 (Gα_13_) [43], involved in downstream activation of RHOA [49]. The NOS1 signal transduction pathway downstream of CCK1R [50, 51] regulates Ca^2+^ signaling pathways by opening ryanodine receptors and two-pore channels that release calcium from the endoplasmic reticulum and endolysosomes, respectively [52–54]. Other signaling cascades, so far only reported for CCK1R, include the Ca^2+^/calmodulin (CaM) dependent activation of CaMKIV and calcineurin (CaN) – NFAT1 signaling pathways [55–57].

### CCK2R-specific signaling pathways

CCK2R activates EGFR via PKC activated MMP3, which cleaves membrane-attached pro-HBEGF into mature HBEGF [58, 59]. PKC isoforms PKC-β and PKC-η have been reported only downstream of CCK2R signaling [60, 61]. CCKR2 specific activation of β-catenin and E-cadherin is mediated by PAK1 [62, 63], and CCK2R specific modulation of BCL-protein family signaling regulates mitochondrial cytochrome C release [64, 65]. CCK2R, but not CCK1R, is reported to activate MAPK7 [66], an upstream regulator of transcription factors MEF-B,-C and D, and the PKC-η target PRKD2 [60], which enhances nuclear export of HDAC7 thereby relieving transcriptional repression of target genes such as NR4A1 [67].

### Segmentation of the CCKR signaling map into modules

**Fig. 4:**
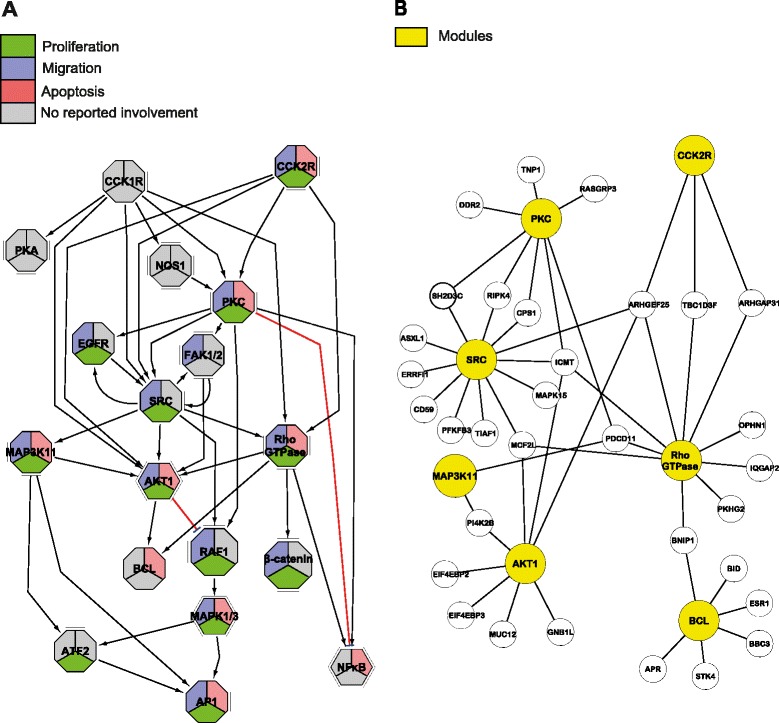
CCKR modular map and PathExpand interactors. **a** The CCKR map modules are connected by ‘activation’ and ‘inhibition’ relationships (indicated by black lines with a pointed arrow and red lines with a bar-headed arrow, respectively) and comprise *i)* receptor-centered modules CCK1R, CCK2R and EGFR, *ii)* modules common to CCK1R and CCK2R (PKC, SRC, MAP3K11, MAPK1/3, RAF1, AKT1, NFκB, MAP3K11, Rho GTPase, FAK1/2), *iii)* CCK1R-specific modules NOS1 and PKA, and *iv)* CCK2R-specific modules BCL and β-catenin. Color-coding depicts published experimentally documented information concerning involvement of the module-encoded signaling mechanisms in gastrin-mediated regulation of cellular responses proliferation, migration and apoptosis. **b** PathExpand interactors (full names, see Table 3) shown for seven of the 18 modules (excluding transcription factor- centered modules)

**Fig. 5:**
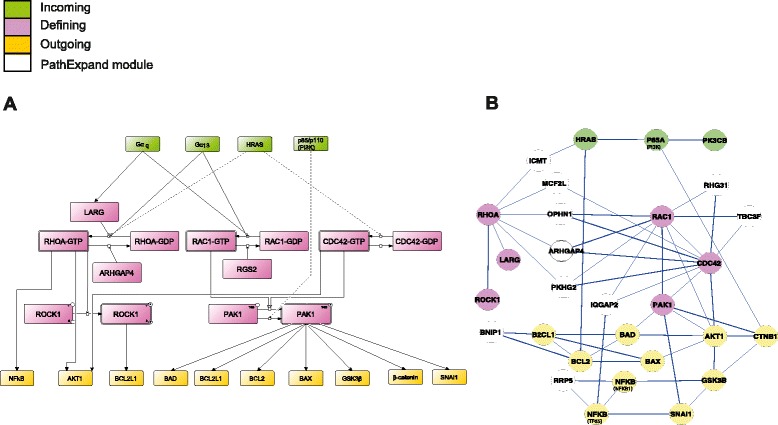
Rho GTPase module and PathExpand interactors. **a** Complete Rho GTPase module including ‘Defining’ components (*pink*) i.e. specific components within this module, as well as ‘Incoming’ (*green*) and ‘Outgoing’ (*yellow*) components representing upstream regulators and downstream effectors respectively. All components shown are encoded in the detailed CCKR map (Fig. 2). **b** Rho GTPase module shown with PathExpand interactors (*colourless*) (Additional file 5)

The complete CCKR signaling map (Fig. 2) is very large. We therefore set out to identify structural and functional subdomains of this map. We used the BiNoM software to import the CCKR map in Cytoscape and built a modular view of the CCKR map similar to what has been previously reported for the E2F-Retinoblastoma signaling pathway [27, 68]. The resulting 18 modules (Methods, Table 2, Fig. 4a, Additional file 2) are fully based on the underlying detailed map and help to comprehend higher order map structure, navigate through the map and work on functional modules. Each of the modules represents a structural and functional signaling subunit, combining a set of closely coordinated molecular events concerning a particular protein or a protein complex (see e.g. the Rho GTPase module in Fig. 5a). Furthermore, the regulatory relations (activating/repressing) connecting the modules (Fig. 4a, black and red arrows) are directly derived from the relations encoded in the underlying detailed map (Fig. 2) and the modular view thus represents central decision-making aspects of CCKR signaling. Additional file 3 gives details for each module including the complete list of components and a discussion of their roles in the signaling mechanisms.

**Table 2:** Overview of the 18 modules and the numbers of defining (specific) components in each module

Module name	# defining components
AKT1	14
AP1	2
ATF2	2
BCL	5
β-catenin	5
CCK1R	4
CCK2R	8
EGFR	3
FAK1/2	6
MAPK1/3	6
MAP3K11	8
NFκB	8
NOS1	8
PKA	10
PKC	12
RAF1	4
Rho GTPase	8
SRC	8

### Gastrin-regulated cellular processes involve different sets of signaling modules

Depending on cell type and the state of cells, gastrin can induce different cellular outcomes, such as proliferation, migration and apoptosis. In order to investigate what is known about the involvement of each of the signaling pathway modules in these processes, we checked the scientific literature that we have used to build the CCKR map. We chose to focus on gastrin-mediated effects, because molecular mechanism studies of gastrin-regulated cellular responses are more abundantly reported in literature. Specifically, we recorded all instances where experimental evidence was reported indicating that the function of a specific signaling component had an effect on the cellular outcome in response to gastrin treatment. Based on this literature survey, involvement in cellular outcomes was indicated in Fig. 4a. While central modules such as PKC, AKT1, Rho GTPase, MAP3K11, MAPK1/3 and AP1 are reportedly involved in all three cellular outcomes, other signaling mechanisms are more specific in their effect, e.g. the BCL-module signaling is only observed to be involved in apoptosis (Fig. 4a). Below, we provide a brief description of signaling modules and components involved in each of the cellular outcomes to gastrin treatment.

### Proliferation

Molecular mechanisms underlying gastrin-mediated proliferation involve regulation of protein synthesis and cell cycle. Protein synthesis is stimulated via the AKT1-module component mTOR triggering p70 S6 kinase [69, 70]. Gastrin-induced transcription of Cyclin D1, a central regulator of cell cycle progression, is mediated by JUN, FOS, CREB1, and TCF7L2 [71–75], which are components of the modules: AP1, ATF2 and β-catenin. The modular representation (Fig. 4a) shows that EGFR-associated signaling enhances gastrin-induced proliferation by feed-forward mechanisms involving SRC module components. The modular map (Fig. 4a) shows that the AKT1 module inhibits RAF1 and consequently MAPK1/3-module pathways. The molecular mechanism for this is AKT1 kinase-mediated phosphorylation of RAF1 [76]. The modular map thus allows us to hypothesize that the activating role of AP1- and ATF2-submodule signaling in proliferation is more likely to proceed via SRC-MAP3K11 pathways, since these are not inhibited by AKT1.

### Migration

Gastrin promotes cell migration by activating transcription of MMP7 and MMP9 [77, 78] via transcription factors SNAI1, β-catenin and JUN [63] represented in the β-catenin- (SNAI1, β-catenin)- and AP1-(JUN) modules. Cell adhesion, tightly linked to cell migration, is regulated through components in the FAK1/2 module (FAK1 and FAK2; Paxillin, CAS, and CRK [79, 80]), which is controlled via both PKC and SRC-modules. The module representation (Fig. 4a) indicates that PKC- and SRC-modules exert a positive feedback regulation on the FAK1/2 module.

### Anti-apoptosis

Apoptosis is inhibited by gastrin via several mechanisms including BCL-mediated repression of pro-apoptotic caspases and AP1-activated expression of Clusterin [64, 65, 81, 82]. The modular representation reveals that these cellular responses are regulated both by PKC independent and PKC dependent mechanisms. This applies to NFκB and its downstream anti-apoptotic *BIRC2* and *BIRC3* target genes, which can be activated either directly by PKC or independently of PKC through the Rho GTPase module. Likewise, the AKT1-involvement in regulation of the BCL-module can be mediated by PKC-dependent mechanisms or independently of PKC by the CCK2R - Rho GTPase pathway. Activation of AP1, on the other hand, seems to be strictly dependent on PKC that mediates its effect via either RAF1-MAPK1/3 or SRC-MAP3K11 cascades.

We note that the AKT1-module both inhibits the RAF1-MAPK1/3-route to AP1-activation and enhances Rho GTPase activation of the BCL-module. Thus, AKT1 can potentially promote BCL-module apoptosis-regulating mechanisms and at the same time block MAPK1/3-mediated AP1-activation. In the latter configuration the cell would rely on MAP3K11 to bypass the inhibitory effect of AKT1 on AP1-mediated regulation of gene expression.

### Extending the CCKR map with PPI data

The comprehensive CCKR signaling map (Fig. 2) has been constructed with a knowledge-driven approach based on molecular reactions and interactions that are well-documented by scientific evidence in the literature. This thereby inevitably leaves significant gaps concerning signaling events and mechanisms that have not yet been studied and evidenced. We have therefore exploited large-scale PPI data to assess how it can complement the CCKR signaling map by a data-driven strategy.

We identified 4119 proteins interacting with at least one CCKR signaling protein (Methods, Additional file 4). Among 199 unique proteins present in our CCKR signal transduction map, 146 were part of this large protein-protein interaction network. We then set out to identify protein interactors that could be of high interest as potential interactors or modulators of the complete CCKR signaling map (Fig. 4a). For this, we analyzed each of the 4119 proteins interacting with the CCKR map proteins using the PathExpand approach [28]. PathExpand checks each interactor to see if it satisfies a number of topological criteria leading to increased compactness of the complete CCKR map. We identified a total of 102 proteins that qualified as PathExpand interactors, 32 of them being components of the original CCKR map. The 70 PathExpand interactors not present in the original CCKR map are listed in Table 3. Interestingly, the set of 70 new CCKR map candidates include 30 proteins that are not known to participate in any signaling pathways listed in the pathway databases KEGG, Reactome, PANTHER and Biocarta (Table 3). A GO term overrepresentation analysis [83] showed that the set of map candidates is enriched in molecular functions relating to protein kinases, protein phosphatases and GTPase-regulators, indicating that many of them could potentially regulate the CCKR pathway via phosphorylation-dephosphorylation mechanisms and by interfering with small GTPase signaling. Among these are the protein kinases STK4, CSK21, CSK22, ITPKA, FLT4, DDR2, KS6KA4 and MAPK15 and a high number of Dual specificity phosphatases (DUSP1, 2, 4, 5, 7, 9, 22) in addition to phosphatases PHLPP1 and PTPRR.

**Table 3:** List of PathExpand interactors

CCK model protein interactors	Protein name	Annotated in pathway databases^a^
AFAP1	actin filament associated protein 1	
AKAP14	A kinase (PRKA) anchor protein 14	
ARHGAP31	Rho GTPase activating protein 31	Yes
ARHGEF25	Rho guanine nucleotide exchange factor (GEF) 25	
ASXL1	additional sex combs like 1 (Drosophila)	
ATP5J	ATP synthase, H+ transporting, mitochondrial Fo complex, subunit F6	Yes
BLVRA	biliverdin reductase A	Yes
BNIP1	BCL2/adenovirus E1B 19 kDa interacting protein 1	Yes
CCDC88A	coiled-coil domain containing 88A	
CD59	CD59 molecule, complement regulatory protein	Yes
CPS1	carbamoyl-phosphate synthase 1, mitochondrial	Yes
DDR2	discoidin domain receptor tyrosine kinase 2	
DUSP2	dual specificity phosphatase 2	Yes
DUSP22	dual specificity phosphatase 22	Yes
DUSP4	dual specificity phosphatase 4	Yes
DUSP5	dual specificity phosphatase 5	Yes
DUSP7	dual specificity phosphatase 7	Yes
DUSP9	dual specificity phosphatase 9	Yes
EIF4EBP2	eukaryotic translation initiation factor 4E binding protein 2	
EIF4EBP3	eukaryotic translation initiation factor 4E binding protein 3	
ERRFI1	ERBB receptor feedback inhibitor 1	
FAM59A	family with sequence similarity 59, member A	
FCGR2C	Fc fragment of IgG, low affinity IIc, receptor for (CD32) (gene/pseudogene)	Yes
FKBP1A	FK506 binding protein 1A, 12 kDa	
FLT4	fms-related tyrosine kinase 4	Yes
GAB3	GRB2-associated binding protein 3	Yes
GJB1	gap junction protein, beta 1, 32 kDa	Yes
GLTSCR1	glioma tumor suppressor candidate region gene 1	
GNB1L	guanine nucleotide binding protein (G protein), beta polypeptide 1-like	
GUCY1A3	guanylate cyclase 1, soluble, alpha 3	Yes
ICMT	isoprenylcysteine carboxyl methyltransferase	
IQGAP2	IQ motif containing GTPase activating protein 2	
IRF5	interferon regulatory factor 5	Yes
ITPKA	inositol-trisphosphate 3-kinase A	Yes
KIAA2026	KIAA2026	
KSR1	kinase suppressor of ras 1	
MAPK15	mitogen-activated protein kinase 15	Yes
MCF2L	MCF.2 cell line derived transforming sequence-like	Yes
MTPN	myotrophin	
MUC12	mucin 12, cell surface associated	
NCF1B	neutrophil cytosolic factor 1B pseudogene	
NFATC3	nuclear factor of activated T-cells, cytoplasmic, calcineurin-dependent 3	Yes
NFKBIZ	nuclear factor of kappa light polypeptide gene enhancer in B-cells inhibitor, zeta	
OGN	osteoglycin	
OPHN1	oligophrenin 1	Yes
PARD6G	par-6 partitioning defective 6 homolog gamma (C. elegans)	Yes
PDCD11	programmed cell death 11	
PDE3B	phosphodiesterase 3B, cGMP-inhibited	Yes
PEBP4	phosphatidylethanolamine-binding protein 4	Yes
PFKFB3	6-phosphofructo-2-kinase/fructose-2,6-biphosphatase 3	Yes
PHLPP1	PH domain and leucine rich repeat protein phosphatase 1	Yes
PI4K2B	phosphatidylinositol 4-kinase type 2 beta	
PIK3C2B	phosphatidylinositol-4-phosphate 3-kinase, catalytic subunit type 2 beta	Yes
PLEKHG2	pleckstrin homology domain containing, family G (with RhoGef domain) member 2	Yes
PPP1R14A	protein phosphatase 1, regulatory (inhibitor) subunit 14A	Yes
PTPN7	protein tyrosine phosphatase, non-receptor type 7	Yes
PTPRR	protein tyrosine phosphatase, receptor type, R	Yes
RASGRP3	RAS guanyl releasing protein 3 (calcium and DAG-regulated)	Yes
RET	ret proto-oncogene	Yes
RIPK4	receptor-interacting serine-threonine kinase 4	
RPL27	ribosomal protein L27	Yes
RPS6KA4	ribosomal protein S6 kinase, 90 kDa, polypeptide 4	Yes
SEMG2	semenogelin II	
SH2D3C	SH2 domain containing 3C	
SIT1	signaling threshold regulating transmembrane adaptor 1	
TBC1D3F	TBC1 domain family, member 3 F	
TIAF1	TGFB1-induced anti-apoptotic factor 1	
TNP1	transition protein 1 (during histone to protamine replacement)	
TPX2	TPX2, microtubule-associated, homolog (Xenopus laevis)	Yes
TRH	thyrotropin-releasing hormone	

^a^includes KEGG, PANTHER, Biocarta and Reactome

Inspection of the expanded networks resulting from the combination of PathExpand protein interactors with 7 CCKR modules (Fig. 4b, transcription factor centered modules excluded) shows that 11 of the protein interactors are linked to more than one CCKR module and can contribute both to PKC-independent (e.g. ARHGEF25, ARHGAP31, TBC1D3F) and PKC dependent (e.g. ICMT) signaling routes. In contrast, 21 protein interactors are linked to only one module, suggesting that they may act as preferential regulators of this module. For instance, the Mitogen activated kinase 15 (MAPK15) is a compactness increasing protein only for the SRC module, where it interacts with the two kinases SRC and CSK.

### CCKR map and genome-scale PPI generate hypotheses for refinement of Rho GTPase module mechanisms

By taking a closer look at the gastrin regulated Rho GTPase module and its identified PPI extensions, we here discuss putative novel signaling mechanisms involved in gastrin-mediated regulation of the Rho GTPase signaling.

Central to the Rho GTPase module (Fig. 5a) is the activation of small GTPases, RHOA, RAC1, and CDC42 by guanine exchange factor proteins (GEFs), which trigger conversion of the inactive GDP-bound form of small GTPases to the active GTP-bound form. The GEFs in gastrin-mediated Rho GTPase signaling are Leukemia-associated Rho guanine-nucleotide exchange factor (LARG) and the trimeric receptor-associated G-protein complexes Gα_q_ and Gα_13_. In addition, HRAS may also act to activate RHOA and CDC42. The small GTPases RHOA, RAC1, and CDC42 are documented to activate kinases ROCK1 and PAK1, which are both part of the gastrin-regulated Rho GTPase module, and of kinase AKT1, part of the gastrin-regulated AKT1 signaling module. The inactive form of the small GTPases is restored by GTPase-activating proteins (GAPs) that enhance hydrolyzation of the bound GTP. In gastrin-mediated signaling deactivation of RHOA and RAC1 is effectuated by GAPs, ARHGAP4 and RGS2, respectively.

Eight of the ten PathExpand protein interactors (Fig. 5b, Additional file 5) of Rho GTPase module components interact with one or more of the small GTPases RHOA, RAC1, and CDC42. All of these protein interactors are annotated with GO-terms indicating a known role in regulation of small GTPases, and four of them (IQGAP2, TBC1D3F, ARHGAP31, OPHN1) are known as GTPase activators. We suggest that these eight proteins are strong candidates for potentially novel modulators of gastrin-mediated Rho GTPase module signaling. Three of the proteins (TBC1D3F, ARHGAP25, and ICMT) are not yet present in any of the common pathway databases (see Table 3). The evidence presented here, indicating that the three proteins have tight interaction with multiple proteins involved in gastrin signaling, demonstrates that our approach is well suited to identify testable hypotheses, even for candidates for which knowledge in public databases is still sparse.

The PathExpand interactor BNIP1 (BCL2/adenovirus E1B 19 kDa interacting protein 1) interacts with two downstream BCL module components BCL2L1 and BCL2, both involved in effectuating the gastrin anti-apoptotic signal. Since BNIP1 is known to be involved in SNARE vesicular transport, our findings indicate that it may be an interesting candidate to follow up with investigating a potential role of vesicular transport in modulating BCL-linked signaling mechanisms in the gastrin signaling pathway. The PathExpand interactor programmed cell death 11 (PDCD11) interacting with the two subunits of NFκB transcription factor (NFκB1 and TF65) is known to be involved in maturation of ribosomal RNA but may also play a role in mRNA processing. Our results identify PDCD11 as a potential candidate to follow up in experimental analysis of molecular mechanisms involved in NFKB-mediated gene regulation in cellular responses to gastrin.

## Discussion

In the work presented here, we set out to build a comprehensive and well-annotated molecular interaction map to aid future studies involving gastrin or CCK. A mechanistic understanding of CCK1R and CCK2R signaling networks is essential for experiment design and data interpretation in biological systems involving gastrin- and CCK-regulated processes. Moreover, it may enhance the identification of therapeutic chemicals able to target disease, by using the map as a functional interaction diagram with components that modeling indicates as prime targets for perturbation, as described by Lee *et al*. [84], who successful designed a drug-induced rewiring of the ‘state’ of oncogenic signaling networks to maximize the susceptibility to anticancer drugs. The current map depicting molecular signaling mechanisms underlying CCK1R and CCK2R triggered cellular responses may provide a tool to guide further investigations into normo- and pathophysiological processes such as gastrin-regulated stomach mucosa homeostatis and gastrin-linked carcinogenesis [59, 64, 85, 86], and cholecystokinin-induced hypoplasia, cell regeneration and digestive enzyme secretion [87] as well as to identify potential intervention strategies for associated disease states.

Some pathway proteins and reactions of the CCKR signaling map are also described in the Reactome [14] and KEGG [15] databases. However, the CCKR map constitutes a vastly more comprehensive and integrated model, providing detailed signaling reactions linking the receptors CCK1R and CCK2R all the way to regulated genes and cellular responses. For comparison, the recently published gastrin signaling map [26] comprises approximately half of the molecules and only a quarter of the reactions presented in our CCKR map. Similarly, knowledge currently in Reactome covers less than 5 % of the pathway details presented here. The CCKR map therefore represents a significant increase in curated signaling information. The provided SBML version of the CCKR map can serve as a starting point to generate quantitative mathematical models [88] for simulation and prediction of cellular outcomes in response to perturbations of the network.

We enhance the applicability of the map for hypothesis generation by two central strategies. First, we provide a computationally modularized version of topologically and functionally connected meta-nodes. This modular view simplifies the complete CCKR map and provides for an improved, higher level comprehension of pathway regulatory aspects concerning cell fate decisions related to proliferation, migration and apoptosis. Secondly, we take advantage of public large scale PPI knowledge to predict new potential regulators of CCKR signaling, including 70 interactors that significantly enhance the compactness of the CCKR map [28], through tight direct and indirect interactions with map proteins. Although experimental validations are needed to confirm these new CCKR signaling mechanisms, they represent an important source of high quality hypotheses that may be taken as a first step to develop a better comprehension of CCKR pathway functionality.

Compared to other recently published computational approaches for high-throughput hypothesis generation [20, 89, 90], the advantage of our strategy is the combination of i) biological background knowledge encoded in the signaling map, including the modules, manually curated from literature reporting detailed experimental analyses of gastrin- and CCK-signaling, and ii) large-scale PPI information downloaded from available databases of interactions, filtered for binary physical interaction based on selected experimental detection methods.

## Conclusion

Our work demonstrates how publicly available tools can aid in the manual curation of an extensive network topology, in order to build a foundation for a systems understanding of CCKR-mediated cellular responses. Furthermore, the integration of a comprehensive model of complex biological networks with genome scale data can provide new knowledge and hypotheses on molecular mechanisms underlying cellular processes. Further development of the resources presented here should be of high interest in translational research aimed at identifying new targets and biomarkers for treatment and diagnostics of gastrin- and/or cholecystokinin-related disease, including cancer.

## Additional files

Additional file 1:
**The file is the SBML version of the complete CCKR map.**
Additional file 2:
**This file is an original Cytoscape session file containing the BiNoM generated modules of the CCKR map.**
Additional file 3:
**The file contains a description of individual modules of the comprehensive CCKR map constructed using the BiNoM plugin in Cytoscape.**
Additional file 4:
**The file contains a list of CCKR model protein interactors identified from the large scale protein-protein interaction network.**
Additional file 5:
**The file contains the PathExpand analysis of BiNoM modules and their protein interactors.**
