# Supplementary material for: The gastrin and cholecystokinin receptors mediated signaling network: a scaffold for data analysis and new hypotheses on regulatory mechanisms
Source: BMC Syst Biol. 2015 Jul 24;9:40. doi: 10.1186/s12918-015-0181-z (PMC4513977; doi:10.1186/s12918-015-0181-z)
Supplement: Additional file 3: — The file contains a description of individual modules of the comprehensive CCKR map constructed using the BiNoM plugin in Cytoscape. [file 12918_2015_181_MOESM3_ESM.pdf]

Additional Information

Description of BiNoM segmented modules

AKT1 module

AKT1 module encompasses components and reaction involved in activation of AKT1-mTOR cascade downstream of the CCKR (Table 1). Both gastrin and CCK activate PI3K via SRC dependent mechanism [1, 2]. Our model shows that gastrin activates tyrosine phosphorylation of IRS1 and its association with p85 subunit of PI3K by recruiting p85/p110 complex at the plasma membrane [3, 4]. Association of IRS1 with p85 activates PI3K complex. CCK2R stimulated JAK2 is documented to function upstream of PI3K in regulation of cell adhesion [5]. Active PI3K triggers PI3K dependent cascade by catalyzing PIP2 into PIP3. Activation of PI3K cascade promotes the recruitment of proteins with pleckstrin homology (PH) domains such as AKT1 and PDPK1 to the plasma membrane. Upon binding to the membrane, AKT1 and PDPK1 become active. Notably, translocation of AKT1 to the plasma membrane also facilitates its phosphorylation by PDPK1 [6, 7]. This cascade of events phosphorylates AKT1 at Ser308 and Ser473 to make it active. It is likely, that activated AKT1 regulates mTOR pathway and stimulates activation of Ribosomal protein S6 kinases 70kDa (p70 S6 kinase) because mTORC1 and PI3K specific inhibitor rapamycin, inhibits gastrin dependent p70S6K activity [8].

Table 1

Module_AKT1								
Incoming			Defining			Outgoing		
components	UniProt accession	UniProt KB entry	components	UniProt accession	UniProt KB entry	components	UniProt accession	UniProt KB entry
Gαq	P29992	GNA11_HUMAN	PRAS40	Q96B36	AKTS1_HUMAN	FOXO1	Q12778	FOXO1_HUMAN
SRC	P12931	SRC_HUMAN	IRS1	P35568	IRS1_HUMAN	FOXO3	O43524	FOXO3_HUMAN
FAK1	Q05397	FAK1_HUMAN	PDPK1	O15530	PDPK1_HUMAN	BAD	Q92934	BAD_HUMAN
CDC42	P60953	CDC42_HUMAN	p70S6K1	P23443	KS6B1_HUMAN	RPS6	P62753	RS6_HUMAN
RhoA	P61586	RHOA_HUMAN	mLST8	Q9BVC4	LST8_HUMAN	4E-BP1	Q13541	4EBP1_HUMAN
SHP2	Q06124	PTN11_HUMAN	mTOR	P42345	MTOR_HUMAN	EIF4E	P06730	IF4E_HUMAN
HRAS	P01112	RASH_HUMAN	PTEN	P60484	PTEN_HUMAN			
p38MAPK	Q16539	MK14_HUMAN	RHEB	Q15382	RHEB_HUMAN			
			RICTOR	Q6R327	RICTR_HUMAN			
			RPTOR	Q8N122	RPTOR_HUMAN			
			JAK2	O60674	JAK2_HUMAN			
			p85	P27986	P85A_HUMAN			
			p110	P42338	PK3CB_HUMAN			
			AKT1	P31749	AKT1_HUMAN			

AP1 module

AP1 module represents the life cycle of members of the AP1 transcription factor, JUN and FOS. Defining members of the AP1 module are listed in Table 2. Gastrin regulates JUN and FOS at both transcriptional and post-translational level. Gastrin mediated JUN gene transcription involves AP1 transcription factor whereas transcription of FOS gene is by MAPK dependent activation of ELK1

transcription factor [9]. At protein level, MAPK8 phosphorylates S63 and S73 residues of the JUN to make it active whereas ATF2 and MAPK1/3 activate FOS by phosphorylating its serine residues [9-11]. Active JUN and FOS protein translocate into the nucleus, associate together and form an AP1 complex.

Table 2

Module_AP1					
**Incoming**			**Defining**		
**components**	**UniProt accession**	**UniProt KB entry**	**components**	**UniProt accession**	**UniProt KB entry**
MAPK8	P45983	MK08_HUMAN	FOS	P01100	FOS_HUMAN
MAPK9	P45984	MK09_HUMAN	JUN	P05412	JUN_HUMAN
MAPK10	P53779	MK10_HUMAN			
p38MAPK	Q16539	MK14_HUMAN			
MAPK3	P27361	MK03_HUMAN			
MAPK1	P28482	MK01_HUMAN			

ATF2 module

This module explains the life cycle of ATF2 transcription factor. Members of the ATF2 module are listed in Table 3. Both MAPK8 and p38MAPK are associated with gastrin dependent activation of ATF2 by phosphorylating its threonine residues [9, 12, 13]. Active ATF2 forms a homodimer and translocates into the nucleus.Nuclear ATF2 associates with JUN to form a complex which regulates the transcription of JUN target gene [9].

Table 3

Module_ATF2					
**Incoming**			**Defining**		
**components**	**UniProt accession**	**UniProt KB entry**	**components**	**UniProt accession**	**UniProt KB entry**
MAPK8	P45983	MK08_HUMAN	ATF2	P15336	ATF2_HUMAN
MAPK9	P45984	MK09_HUMAN	JUN	P05412	JUN_HUMAN
MAPK10	P53779	MK10_HUMAN			
p38MAPK	Q16539	MK14_HUMAN			

BCL module

The BCL module includes BCL2 family proteins associated with gastrin dependent apoptosis regulation mechanism. In the comprehensive CCKR map, BCL2 family circumscribes both pro-apoptotic (BAX and BAD) and anti-apoptotic (BCL2, BCL2L1 and MCL1) members (Table 4). Gastrin activates expression of BCL2 and BCL2L1 via Rho GTPase dependent mechanism [14], whereas expression of MCL1 is mediated via AP1 dependent pathway [15]. Rho GTPase dependent activation of target proteins ROCK1 and PAK1 influences the expression of BCL2 and BCL2L1 proteins. Oligomerization of the BAX proteins causes release of cytochrome C from mitochondria, as a consequence activation of caspase 3. The BCL2-like proteins form heterodimers with BAX or BAD

which results in the inhibition of the release of cytochrome C from the mitochondria [14, 16, 17]. Gastrin activates PAK1 and AKT1 proteins which then phosphorylate BAD at Ser136 and Ser112 residues, resulting in the dissociation of BAD from its heterodimer partners, BCL2 and BCL2L1 [14, 18, 19]. Dissociation of BAD stops release of cytochrome c from the mitochondria, as a consequence inhibits caspase 3 activation [14]. Gastrin inactivates both FOXO1 and FOXO3 transcription factors by AKT1 dependent phosphorylation [19] resulting in the inhibition of apoptosis.

Table 4

**Module_BCL**								
**Incoming**			**Defining**			**Outgoing**		
**components**	**UniProt accession**	**UniProt KB entry**	**components**	**UniProt accession**	**UniProt KB entry**	**components**	**UniProt accession**	**UniProt KB entry**
PAK1	Q13153	PAK1_HUMAN	BCL2L1	Q07817	B2CL1_HUMAN	Caspase 3	P42574	CASP3_HUMAN
ROCK1	Q13464	ROCK1_HUMAN	BAD	Q92934	BAD_HUMAN			
AKT1	P31749	AKT1_HUMAN	BAX	Q07812	BAX_HUMAN			
			BCL2	P10415	BCL2_HUMAN			
			MCL1	Q07820	MCL1_HUMAN			

Beta-catenin module

Beta-catenin module circumscribes components and reactions associated with gastrin dependent beta-catenin/E-cadherin interaction (Table 5). Beta catenin and E-cadherin form an adhesion complex at the membrane. This adhesion complex is disrupted by gastrin to promote cell migration and invasion. Gastrin activated PAK1 phosphorylates SNAI1 transcription factor at Ser246. Consequently, SNAI1 translocates to the nucleus and inhibits E-cadherin gene transcription [20, 21]. As a result, number of E-cadherin molecules present at the membrane decreases. Decrease in E-cadherin molecule causes disruption of Beta-catenin/E-cadherin interaction [21]. Now, intact Beta-catenin translocates into the cytoplasm and undergoes phosphorylation at Ser45 mainly by caseine kinase1 (CK1). Importantly, cytosolic phosphorylated beta-catenin may undergo GSK3beta dependent phosphorylation and eventual degradation but gastrin activated PAK1 prevent this degradation by inactivating GSK3beta. PAK1 inactivates GSK3beta by phosphorylating its Ser9 residue [20, 21]. Furthermore, PAK1 triggers nuclear transport of the activate beta-catenin [20, 21]. Nuclear beta-catenin associates with transcription factor TCF7L2 and regulate expression of several target genes in response to gastrin.

Table 5

**Module_Beta-catenin**								
**Incoming**			**Defining**			**Outgoing**		
**components**	**UniProt accession**	**UniProt KB entry**	**components**	**UniProt accession**	**UniProt KB entry**	**components**	**UniProt accession**	**UniProt KB entry**
PAK1	Q13153	PAK1_HUMAN	E-cadherin	P12830	CADH1_HUMAN	TCF7L2	Q9NQB0	TF7L2_HUMAN
			Beta-catenin	P35222	CTNB1_HUMAN			
			GSK3 beta	P49841	GSK3B_HUMAN			
			SNAI1	O95863	SNAI1_HUMAN			
			CK1	P48729	KC1A_HUMAN			

CCK1R module

The CCK1R module depicts the life cycle of the CCK1 receptor. Members of the CCK1R module are listed in Table 6. Sulfated CCK binds to active CCK1R and triggers downstream signaling cascades. Under CCK stimulation, CCK1R is rapidly phosphorylated at consensus serine residues in the third intracellular loop, both by PKC and a G protein kinase, causing receptor inactivation [22, 23]. Desensitization and further recycling of the CCK1R happens by receptor-endocytosis in the cytosol. After stimulation of CCK1R by CCK, ligand bound receptor complex is internalized into an endocytic vesicle [24, 25]. From the endosome, CCK and CCK1R are then sorted into their destined cellular location. Notably, average sorting time of CCK and receptor in endosome is about 25 minutes. CCK is sorted into the lysosome and undergoes proteosomal degradation whereas the receptor recycles back to the cell membrane with an average time of 60 min [25].

Table 6

Module_CCK1R								
Incoming			Defining			Outgoing		
components	UniProt accession	UniProt KB entry	components	UniProt accession	UniProt KB entry	components	UniProt accession	UniProt KB entry
CCK	P06307	CCKN_HUMAN	CCK1R	P32238	CCKAR_HUMAN	MAPK8	P45983	MK08_HUMAN
			Gαq	P29992	GNA11_HUMAN	MAPK9	P45984	MK09_HUMAN
			Gαs	P63092	GNAS2_HUMAN	MAPK10	P53779	MK10_HUMAN
			Gα13	Q14344	GNA13_HUMAN	p38MAPK	Q16539	MK14_HUMAN
						SHP2	Q06124	PTN11_HUMAN
						CD38	P28907	CD38_HUMAN

EGFR module

EGFR module encompasses components associated with the activation of EGFR (Table 7). Gastrin induces expression of HB-EGF and EGFR transactivation as documented in human gastric cancer cell line [26] and in rat gastric epithelial cells [27, 28]. CCK2R activates matrix metalloproteinase 3 (MMP-3) via PKC dependent mechanism [28]. Activated MMP-3 cleaves Glu-Asn site within the juxtamembrane (JM) region of the membrane anchored pro-HBEGF into soluble mature HBEGF [26, 27]. Mature HBEGF then binds to the EGFR and activates this receptor by phosphorylating several tyrosine residues. Both SRC and SHC1 associate with active EGFR. SHC1 binds to the tyrosine residues 1148/1173 of the active EGFR. EGFR activates SHC1 by phosphorylating its Y317 residue [29]. Also, GRB2 transports from cytosol and binds to the active EGFR receptor at membrane on either phosphorylated Y1068 or Y1086 [30]. This binding recruits SOS1 onto the membrane which forms a complex with GRB2. Phosphorylated SHC1 associates with GRB2/SOS1 complex and regulate CCK2R dependent MAPK cascade [31, 32].

Table 7

Module_EGFR								
Incoming			Defining			Outgoing		
components	UniProt accession	UniProt KB entry	components	UniProt accession	UniProt KB entry	components	UniProt accession	UniProt KB entry
SRC	P12931	SRC_HUMAN	EGFR	P00533	EGFR_HUMAN	GRB2	P62993	GRB2_HUMAN
			MMP3	P08254	MMP3_HUMAN	SOS1	Q07889	SOS1_HUMAN
			HBEGF	Q99075	HBEGF_HUMAN	SHC1	P29353	SHC1_HUMAN

CCK2R module

CCK2R represents the life cycle of CCK2 receptor. Members of the CCK2R module are listed in Table 8. Amidated gastrin binds to the CCK2R, which leads to the phosphorylation and activation of CCK2R. Active ligand-receptor complex triggers downstream signaling cascades. Internalization and intracellular trafficking of the CCK2R primarily involves binding of beta-arrestin adaptor proteins to the receptor and clathrin coated pits. After CCK2R stimulation by gastrin, beta-arrestin ½ transports from cytoplasm to the plasma membrane where it interacts with C-terminal phosphorylated residues of the CCK2 receptor [33]. Beta-arrestin bound CCK2R is then recuited into clathrin-coated endocytic vesicle. Interestingly, gastrin too found to be trapped into this endocytic vesicle but without any clear evidence whether it remains intact with the receptor or they were degraded by proteases [33]. It was examined that CCK2R internalization is also dependent on the activity of a GTPase, dynamin. Dynamin acts as a mechanochemical enzyme to clip membrane attached vesicles and their targeting, fusion with another compartment. Furthermore, internalized CCK2R does not recycle rapidly to the cell surface. Instead, CCK2R directs to the late endosome/lysosome, indicating a possibility of slow recycling/degradation.

Table 8

**Module_CCK2R**								
**Incoming**			**Defining**			**Outgoing**		
**components**	**UniProt accession**	**UniProt KB entry**	**components**	**UniProt accession**	**UniProt KB entry**	**components**	**UniProt accession**	**UniProt KB entry**
G17	P01350	GAST_HUMAN	Beta-arrestin-1	P49407	ARRB1_HUMAN	PLCγ1	P19174	PLCG1_HUMAN
CCK	P06307	CCKN_HUMAN	Beta-arrestin-2	P32121	ARRB2_HUMAN	PLCβ	Q9NQ66	PLCB1_HUMAN
			Dynamin	Q05193	DYN1_HUMAN	TRAF6	Q9Y4K3	TRAF6_HUMAN
			RGS2	P41220	RGS2_HUMAN	PLA2	P47712	PA24A_HUMAN
			CCK2R	P32239	GASR_HUMAN	MAP2K5	Q13163	MP2K5_HUMAN
			Gαq	P29992	GNA11_HUMAN	RAC1	P63000	RAC1_HUMAN
			AP2M1	Q96CW1	AP2M1_HUMAN	CDC42	P60953	CDC42_HUMAN
			PLA2	P47712	PA24A_HUMAN	JAK2	O60674	JAK2_HUMAN
						SHP2	Q06124	PTN11_HUMAN
						LARG	Q9NZN5	ARHGC_HUMAN
						SRC	P12931	SRC_HUMAN

FAK1/2 module

Tyrosine phosphorylation and activation of FAK1 is described in response to both CCK1R and CCK2R stimulation while FAK2 activation is recorded only in response to CCK. Gastrin controls cell adhesion by signaling pathways involving FAK1, Paxillin [34], Crk-associated substrate (CAS), and v-crk sarcoma virus CT10 oncogene (CRK) [34-36]. Gastrin activates CAS/CRK complex formation by p60SRC and PKC dependent pathway [36] (detail list in Table 9). Our model represents that FAK1 associates with p60SRC and p190RhoGEF to form a complex. This complex then regulates phosphorylation and activation of paxillin [34]. Interestingly, FAK1-p60SRC complex acts upstream of the gastrin-stimulated PI 3-kinase pathway [2]. In rat pancreatic acinar cells, CCK-8 rapidly stimulates tyrosine phosphorylation and activation FAK2. This activation of FAK2 is mediated by PKC and increase of [Ca^{2+}] [37, 38]. CCK stimulation causes a rapid formation of both FAK2-GRB2 and FAK2-CRK complexes [37]. The exact mechanism of FAK2 activation by Ca^{2+} is still not understood [39], but inhibiting PKC-θ in rat pancreatic acinar cells has been shown to inhibit phosphorylation of Tyr-402 of FAK2 [40], indicating that PKC-θ is the link between CCK1R

stimulation and FAK2 activation. FAK2 auto-phosphorylates at Tyr402. Phosphorylation at Tyr402 provides a binding site for SH2 containing proteins including SRC and p85. Binding of SRC leads to phosphorylation of FAK2 residues Tyr579 and Tyr580, with maximal FAK2 kinase activity [41, 42]. Phosphorylation at Tyr-881 by SRC promotes interaction of FAK2 with GRB2 [42]. FAK2 also forms a complex with CRK in rat pancreatic acinar cells after stimulation with CCK-8 [37]. In rat pancreatic acinar cells CCK also stimulates formation of CRK-CAS complex [43].

Table 9

Module_FAK1/2								
**Incoming**			**Defining**			**Outgoing**		
**components**	**UniProt accession**	**UniProt KB entry**	**components**	**UniProt accession**	**UniProt KB entry**	**components**	**UniProt accession**	**UniProt KB entry**
SRC	P12931	SRC_HUMAN	CAS	P56945	BCAR1_HUMAN	HRAS	P01112	RASH_HUMAN
GRB2	P62993	GRB2_HUMAN	CRK	P46108	CRK_HUMAN			
PKC-θ	Q04759	KPCT_HUMAN	FAK1	Q05397	FAK1_HUMAN			
			FAK2	Q14289	FAK2_HUMAN			
			Paxillin	P49023	PAXI_HUMAN			
			p190RhoGEF	Q8N1W1	RGNEF_HUMAN			

MAPK1/3 module

MAPK1/3 module constitutes cascade of events associated with the activation of MAPK1 (ERK2) and MAPK3 (ERK1) signaling pathway downstream of CCKR (Table 10). CCKR activate MAPK1/3 through RAF dependent stimulation of MAP2K1/2.Activation of RAF is achieved either by GRB2/SOS dependent activation of HRAS or by stimulation of RAF through PKC-mediated mechanisms. Activation of RAF1 is independent of PKC activity in Rat1 cells whereas in human gastric cancer cells, RAS independent activation of RAF is detected in response to gastrin [44, 45].Active RAF1 phosphorylates serine residues of dual specificity kinases, MAP2K1 and MAP2K2. Phosphorylated MAP2K1 and MAP2K2 then activate MAPK1/3 by phosphorylating their threonine/tyrosine residues. Active MAPK1/3 then form a homodimer and transports into the nucleus where they regulate the activity of several TFs and TGs. Active MAPK1/3 also triggers RSK (RSK1/2) activation cascade by phosphorylating its serine/threonine residues [46]. Active RSK translocates into the nucleus where it plays a role in the activation of CREB1 TF by phosphorylating its S133 residue [46, 47]. Our modular view indicates that MAPK1/3 module has positive influence on AP1 and ATF2 modules.

Table 10

Module_MAPK1/3								
**Incoming**			**Defining**			**Outgoing**		
**components**	**UniProt accession**	**UniProt KB entry**	**components**	**UniProt accession**	**UniProt KB entry**	**components**	**UniProt accession**	**UniProt KB entry**
ARAF	P10398	ARAF_HUMAN	MAPK3	P27361	MK03_HUMAN	CREB1	P16220	CREB1_HUMAN
BRAF	P15056	BRAF_HUMAN	RSK1	Q15418	KS6A1_HUMAN	ELK1	P19419	ELK1_HUMAN
RAF1	P04049	RAF1_HUMAN	MAPK1	P28482	MK01_HUMAN	SP1	P08047	SP1_HUMAN
			MAP2K2	P36507	MP2K2_HUMAN	SP3	Q02447	SP3_HUMAN
			RSK2	P51812	KS6A3_HUMAN	SAP1	P28324	ELK4_HUMAN
			MAP2K1	Q02750	MP2K1_HUMAN	EGR1	P18146	EGR1_HUMAN

SRF	P11831	SRF_HUMAN
PPARγ	P37231	PPARG_HUMAN
FOS	P01100	FOS_HUMAN

MAP3K11 (MLK3) module

In the modular view, MAP3K11 module represents components and reactions involved in the activation of MAPK8 (JNK) and p38MAPK (Table 11). Activation of MAPK8 and p38MAPK is reported in response to both CCK1R and CCK2R stimulation by CCK and gastrin respectively. MAP3K11 is a serine/threonine kinase with SH3 domain-containing proline-rich kinase. HRAS seems to be the upstream regulator of MAP3K11. MAP3K11 is a known activator of dual specificity protein kinases MAP2K4 and MAP2K6 by phosphorylating Ser257/Thr261 residues of MAP2K4, and Ser207/Thr211 residues of MAP2K6. Phosphorylated MAP2K4 and MAP2K6 are the upstream regulators of MAPK8 and p38MAPK respectively [36, 48, 49]. MAP2K4 phosphorylates Thr183/Tyr185 residues of MAPK8 whereas MAP2K6 phosphorylates Thr180/Tyr182 residues of p38MAPK. Both MAPK8 and p38MAPK activate transcription factor ATF2 whereas only MAPK8 stimulate transcription factor JUN in RIE-1/CCK2R cells treated with gastrin [48].

Table 11

Module_MAP3K11								
Incoming			Defining			Outgoing		
components	UniProt accession	UniProt KB entry	components	UniProt accession	UniProt KB entry	components	UniProt accession	UniProt KB entry
HRAS	P01112	RASH_HUMAN	MAPK8	P45983	MK08_HUMAN	AKT1	P31749	AKT1_HUMAN
			MAP3K11	Q16584	M3K11_HUMAN	NFkB1	P19838	NFKB1_HUMAN
			p38MAPK	Q16539	MK14_HUMAN	RELA	Q04206	TF65_HUMAN
			MAP2K4	P45985	MP2K4_HUMAN	JUN	P05412	JUN_HUMAN
			MAP2K6	P52564	MP2K6_HUMAN	ATF2	P15336	ATF2_HUMAN
			MAPK9	P45984	MK09_HUMAN	MAPKAP-K2	P49137	MAPK2_HUMAN
			MAPK10	P53779	MK10_HUMAN	MEF2B	Q02080	MEF2B_HUMAN
			MAPKAP-K2	P49137	MAPK2_HUMAN	MEF2C	Q06413	MEF2C_HUMAN
						MEF2D	Q14814	MEF2D_HUMAN

NFκB module

This module encircles CCKR dependent activation mechanism of the NFκB transcription factor (Table 12). CCK1R and CCK2R dependent activation of NFκB is via PKCδ [9, 50]. It has been reported that both PKCδ and PKCε are involved in CCK mediated NFκB activation [50], and our model suggests that PRKD1 could be the possible link between this activation [51]. Gastrin stimulated CCK2R follows TRAF6/TAK1/MAP3K14 pathway to activate NFκB [9]. In this cascade, MAP3K14 which is also known as NFκB inducing kinase (NIK) activates IκB kinase. Activated IκB kinase then phosphorylates S32 and S36 residues of the IκB, as a result releases the inhibitory effect of IκB on the NFκB1-RELA complex. Phosphorylated IκB dissociates from the NFκB1-RELA complex and undergoes proteosomal degradation [9], leaving active NFκB1-RELA complex which then translocates into the nucleus. Gastrin and cholecystokinin promote NFκB nuclear translocation via RhoA and MAPK8 dependent pathways respectively [52, 53]. In CCK1R system, it has been observed that PKC-α exerts an inhibitory effect on NFκB activation in rat pancreatic acini [50].

Table 12

Module_NFκB					
**Incoming**			**Defining**		
**components**	**UniProt accession**	**UniProt KB entry**	**components**	**UniProt accession**	**UniProt KB entry**
PKCα	P17252	KPCA_HUMAN	IKKα	O15111	IKKA_HUMAN
PKCδ	Q05655	KPCD_HUMAN	TRAF6	Q9Y4K3	TRAF6_HUMAN
PKCε	Q02156	KPCE_HUMAN	MAP3K14	Q99558	M3K14_HUMAN
RhoA	P61586	RHOA_HUMAN	IKKγ	Q9Y6K9	NEMO_HUMAN
PRKD1	Q15139	KPCD1_HUMAN	NFKB1	P19838	NFKB1_HUMAN
			TAK1	P49116	NR2C2_HUMAN
			IKKβ	O14920	IKKB_HUMAN
			RELA	Q04206	TF65_HUMAN

NOS1 module

The nitric oxide-cGMP pathway is known to be activated by CCK in rat pancreatic acinar cells [54] and in the CHO cell line by CCK1R [55-57]. Memebers of the NOS1 module are listed in Table 13. The link between CCK1R and nitric oxide synthase (NOS1) is still unknown for pancreatic acinar cells, but it is shown that neuronal NOS1 (nNOS) is activated by the Gβγ-subunit and activated tyrosine phosphatase SHP-2 in CHO cells. SHP-2 associated with the Gβ1 subunit, became activated, and then dephosphorylated nNOS through direct association [56].
Activated NOS1 cleaves L-arginine, forming L-citrulline and NO. NO then activates soluble guanylate cyclase [56], which produces cGMP from GMP. cGMP then (directly or via other components) activates a cytosolic ADP-ribosyl cyclase (CD38). This CD38 produces cyclic cADPr from NAD^+ [58]. cADPr then activates ryanodine receptor (RyR) in the endoplasmic reticulum, which then facilitates the transport of Ca^{2+} from the ER to the cytosol [59]. RyR is shown to be active in signaling in pancreatic acinar cells [60]. The Ca^{2+}-induced Ca^{2+} release (CICR) mechanism enhances calcium transport from ER to cytosol, and is also mediated by the ryanodine receptor [59].
Ryanodine receptors consist of three isoforms, RYR1, RYR2 and RYR3, and all three isoforms are expressed in rat pancreatic tissue, with RYR1 and RYR2 specifically found in pancreatic acinar cells [61]. Ca^{2+} is thus increased by both the IP3 and cADPr pathway, and it has been shown in mouse pancreatic acinar cells that CCK-induced Ca^{2+}-spiking can be mediated by both pathways, and that the pathway mediating the response is dependent on intracellular glucose levels: High glucose levels potentiates IP3-evoked Ca^{2+}-spiking, and low glucose levels potentiates cADPR-evoked Ca^{2+}-spiking [62].

Table 13

Module_NOS1					
**Incoming**			**Defining**		
**components**	**UniProt accession**	**UniProt KB entry**	**components**	**UniProt accession**	**UniProt KB entry**
SHP2	Q06124	PTN11_HUMAN	CD38	P28907	CD38_HUMAN

NOS1	P29475	NOS1_HUMAN
Guanylate cyclase	Q02846	GUC2D_HUMAN
RYR1	P21817	RYR1_HUMAN
RYR2	Q92736	RYR2_HUMAN
RYR3	Q15413	RYR3_HUMAN
cGK 1	Q13976	KGP1_HUMAN
SHP2	Q06124	PTN11_HUMAN

PKA module

In the modular view, CCK1R module manifests positive influence on the PKA module (list of components in Table 14). CCK1R is coupled to G_S and CCK1R stimulation then activates adenylate cyclase [63]. Active adenylate cyclase converts ATP into cAMP which then activates cAMP-dependent protein kinase (cAPK)/PKA by releasing the catalytic subunits from the regulatory subunits [64]. PKA is a heterotetramer in its inactive form, with two regulatory subunits binding the catalytic subunits. Different subunits have different affinities for cAMP, generating holoenzymes (PKA type I or type II). Each regulatory subunit binds two cAMP molecules, releasing the catalytic subunits[65]. PKA then phosphorylates serine and threonine residues on specific substrate proteins both in the cytoplasm and in the nucleus [66]. The catalytic subunits of PKA translocates to the cell nucleus, where the transcript factor CREB is activated through phosphorylation [64].

Table 14

**Module_PKA**								
**Incoming**			**Defining**			**Outgoing**		
**components**	**UniProt accession**	**UniProt KB entry**	**components**	**UniProt accession**	**UniProt KB entry**	**components**	**UniProt accession**	**UniProt KB entry**
Gαs	P63092	GNAS2_HUMAN	Adenylate cyclase	Q08828	ADCY1_HUMAN	CREB1	P16220	CREB1_HUMAN
			AKAP	Q92667	AKAP1_HUMAN	IP3R	Q14643	ITPR1_HUMAN
			PKA-R2α	P13861	KAP2_HUMAN			
			PKA-Cα	P17612	KAPCA_HUMAN			
			PKA-Cβ	P22694	KAPCB_HUMAN			
			PDE	P54750	PDE1A_HUMAN			
			PKA-Cγ	P22612	KAPCG_HUMAN			
			PKA-R1α	P10644	KAP0_HUMAN			
			PKA-R1β	P31321	KAP1_HUMAN			
			PKA-R2β	P31323	KAPCG_HUMAN			

PKC module

In the comprehensive map, PKC module depicts activation of different members of the PKC family (detail list in Table 15). CCKR elicits DAG and IP3 production via PLC dependent mechanism by catalyzing PIP2. In CCK2R system, both PLCβ and PLCγ1 dependent IP3 production has been documented while in CCK1R system only PLCβ mediated DAG and IP3 production is reported. Activated IP3 then binds to IP3 receptor in the ER and triggers oscillation of Ca^{2+} from ER to cytosol. PKC superfamily has 3 different subfamilies: i) conventional PKCs, members of this family require both DAG and Ca^{2+} for activation. PKCα and PKCβ are the members of this family which are present

in our model, ii) novel PKCs, members of this family are DAG responsive and Ca^{2+} unresponsive. PKCδ, PKCϵ, PKCη, and PKCθ are the members of this family, and iii) atypical PKCs, members of this family require neither DAG nor Ca^{2+} for activation. PKCζ is a member of this family which has been reported to play a role in CCKR signaling. Active PKCs are involved in activation of another serine/threonine kinase, protein kinase D (PRKD). Both CCK1R and CCK2R dependent activation of PRKD1 has been established while activation of PRKD2 is documented only downstream of CCK2R. The specific PKC isoforms associated with PRKD1 activation after CCK stimulation are PKCδ, PKCϵ, and PKCθ [40, 51].

In human gastric cancer cells stably transfected with the CCKB/gastrin receptor , gastrin stimulates PRKD2 activation by PKC-α, -ε, and -η dependent phosphorylation of its residues (Sturany, Van Lint et al. 2001; Sturany, Van Lint et al. 2002; von Blume, Knippschild et al. 2007). Phosphorylation of PRKD2 at Ser244 within the zinc-finger domain by Casein Kinase (CK1)-δ and -ε promotes nuclear accumulation of PRKD2 in response to gastrin (von Blume, Knippschild et al. 2007).

Table 15

Module_PKC								
Incoming			Defining			Outgoing		
components	UniProt accession	UniProt KB entry	components	UniProt accession	UniProt KB entry	components	UniProt accession	UniProt KB entry
PKA-Cα	P17612	KAPCA_HUMAN	PRKD1	Q15139	KPCD1_HUMAN	SHC1	P29353	SHC1_HUMAN
PKA-Cβ	P22694	KAPCB_HUMAN	PKCδ	Q05655	KPCD_HUMAN	SRC	P12931	SRC_HUMAN
PKA-Cγ	P22612	KAPCG_HUMAN	PKCη	P24723	KPCL_HUMAN	RAF1	P04049	RAF1_HUMAN
Gαq	P29992	GNA11_HUMAN	PKCθ	Q04759	KPCT_HUMAN	MMP3	P08254	MMP3_HUMAN
RYR1	P21817	RYR1_HUMAN	PKCβ	P05771	KPCB_HUMAN	NFkB1	P19838	NFKB1_HUMAN
RYR3	Q15413	RYR2_HUMAN	PRKD2	Q9BZL6	KPCD2_HUMAN	RELA	Q04206	TF65_HUMAN
RYR2	Q92736	RYR3_HUMAN	PKCζ	Q05513	KPCZ_HUMAN	CCK1R	P32238	CCKAR_HUMAN
			PLCβ	Q9NQ66	PLCB1_HUMAN	RhoA	P61586	RHOA_HUMAN
			PKCε	Q02156	KPCE_HUMAN	YES1	P07947	YES_HUMAN
			IP3R	Q14643	ITPR1_HUMAN	LYN	P07948	LYN_HUMAN
			PLCγ1	P19174	PLCG1_HUMAN	HDAC7	Q8WUI4	HDAC7_HUMAN
			PKCα	P17252	KPCA_HUMAN	FAK2	Q14289	FAK2_HUMAN
						ARAF	P10398	ARAF_HUMAN
						HRAS	P01112	RASH_HUMAN

RAF1 module

RAF family constitutes three serine/threonine protein kinases, A-RAF, B-RAF, and C-RAF (RAF1) (Table 16). These protein kinases act as a regulatory link between membrane bound RAS-GTPase and MAPK cascade. CCK1R has been implicated to activate all three RAFs [40, 67] whereas only RAF1 is documented to be activated in response to gastrin [44]. RAF1 module mainly represents life cycle of the RAF1 activation. Active HRAS dissociates RAF1 from the RAF1-14-3-3 complex and then recruits it to the plasma membrane from the cytosol [68]. Sequential phosphorylation of serine/threonine/tyrosine (except S259) residues of the membrane attached RAF1 by different kinases results into an active RAF1. RAF1 activates MAPK1/3 cascade by triggering phosphorylation of MAP2K1/2 proteins. Active AKT1 inactivates RAF1 by phosphorylating its S259 residue [69].

Table 16

Module_RAF1								
Incoming			Defining			Outgoing		
components	UniProt accession	UniProt KB entry	components	UniProt accession	UniProt KB entry	components	UniProt accession	UniProt KB entry
HRAS	P01112	RASH_HUMAN	14-3-3β/α	P31946	1433B_HUMAN	MAP2K1	Q02750	MP2K1_HUMAN
PKCθ	Q04759	KPCT_HUMAN	ARAF	P10398	ARAF_HUMAN	MAP2K2	P36507	MP2K2_HUMAN
AKT1	P31749	AKT1_HUMAN	BRAF	P15056	BRAF_HUMAN			
			RAF1	P04049	RAF1_HUMAN			

Rho GTPase module

The Rho GTPase module represents components and reactions involved in the activation of members of the Rho GTPase family (Table 17). Members of this family include: RHOA, RAC1, and CDC42. Both gastrin and cholecystokinin can activate RHOA and RAC1, while only gastrin is reported to be involved in the activation of CDC42 [14]. CCK2R mediated activation of Rho GTPases (RHOA, RAC1 and CDC42) from the inactive GDP-bound form to the active GTP-bound form is via $G\alpha_q$. Guanine exchange factors (GEFs), for example Leukemia-associated Rho guanine-nucleotide exchange factor (LARG) can serve as an effector for $G\alpha_q$ – coupled receptors [70] and GTPase-activating proteins (GAPs) hydrolyzes GTP to convert active GTP-bound form of Rho GTPases into inactive GDP-bound form Gastrin-stimulated RHOA acts through interaction with a serine/threonine kinase, ROCK whereas RAC1 and CDC42 acts through specific effector protein, PAK1 [14].

Table 17

Module_RhoGTPase								
Incoming			Defining			Outgoing		
components	UniProt accession	UniProt KB entry	components	UniProt accession	UniProt KB entry	components	UniProt accession	UniProt KB entry
Gαq	P29992	GNA11_HUMAN	PAK1	Q13153	PAK1_HUMAN	AKT1	P31749	AKT1_HUMAN
Gα13	Q14344	GNA13_HUMAN	ROCK1	Q13464	ROCK1_HUMAN	NFkB1	P19838	NFKB1_HUMAN
HRAS	P01112	RASH_HUMAN	CDC42	P60953	CDC42_HUMAN	RELA	Q04206	TF65_HUMAN
p85	P27986	P85A_HUMAN	RAC1	P63000	RAC1_HUMAN	BCL2L1	Q07817	B2CL1_HUMAN
p110	P42338	PK3CB_HUMAN	RHOA	P61586	RHOA_HUMAN	BAD	Q92934	BAD_HUMAN
			LARG	Q9NZN5	ARHGC_HUMAN	BAX	Q07812	BAX_HUMAN
			ARHGAP4	P98171	RHG04_HUMAN	BCL2	P10415	BCL2_HUMAN
			RGS2	P41220	RGS2_HUMAN	GSK3beta	P49841	GSK3B_HUMAN
						beta-catenin	P35222	CTNB1_HUMAN
						SNAI1	O95863	SNAI1_HUMAN

SRC module

This module represents the role of GRB2, SHC, and SRC proteins in the CCKR signaling (detail list in Table 18). Both SRC and SHC proteins are activated by CCKR. PKC dependent phosphorylation of

SHC is reported for both CCK1 and CCK2 receptors [32, 71] while activation of SRC via PKC is documented only in response to gastrin [32]. The SHC-gene (SHC1) encodes three major isoforms of SHC, p46SHC, p52SHC, and p66SHC. Gastrin mediates time and dose dependent increase in tyrosine phosphorylation of p46 SHC and p52SHC isoforms of adaptor protein SHC1 in AR42J cells. Gastrin induced phosphorylation of SHC is dependent on SRC kinase [72] and PKC isoforms (PKC-α,-δ,-ε) [32]. Active SRC, SHC1 associate with ligand bound EGFR. Further, active EGFR associates with GRB2 at the membrane which then recruits SOS1 onto the membrane from the cytosol. SHC1 forms an active complex with GRB2-SOS1 which leads to the activation of HRAS-RAF1-MAPK cascade [31]. GRB2 and SRC are also involved in activation of the FAK2 cascade in response to cholecystokinin [73, 74].

Table 18

**Module_SRC**								
**Incoming**			**Defining**			**Outgoing**		
**components**	**UniProt accession**	**UniProt KB entry**	**components**	**UniProt accession**	**UniProt KB entry**	**components**	**UniProt accession**	**UniProt KB entry**
PKCα	P17252	KPCA_HUMAN	GRB2	P62993	GRB2_HUMAN	PLCγ1	P19174	PLCG1_HUMAN
PKCθ	Q04759	KPCT_HUMAN	SRC	P12931	SRC_HUMAN	FAK1	Q05397	FAK1_HUMAN
Gαq	P29992	GNA11_HUMAN	SOS1	Q07889	SOS1_HUMAN	FAK2	Q14289	FAK2_HUMAN
PKCδ	Q05655	KPCD_HUMAN	HRAS	P01112	RASH_HUMAN	RAF1	P04049	RAF1_HUMAN
PKCε	Q02156	KPCE_HUMAN	CSK	P41240	CSK_HUMAN	ARAF	P10398	ARAF_HUMAN
IRS1	P35568	IRS1_HUMAN	SHC2	P98077	SHC2_HUMAN	BRAF	P15056	BRAF_HUMAN
FAK2	Q14289	FAK2_HUMAN	SHC3	Q92529	SHC3_HUMAN	RhoA	P61586	RHOA_HUMAN
			SHC1	P29353	SHC1_HUMAN	CDC42	P60953	CDC42_HUMAN
						AKT1	P31749	AKT1_HUMAN
						MAP3K11	Q16584	M3K11_HUMAN

D) References

1. Kim M, Nozu F, Kusama K, Imawari M: **Cholecystokinin stimulates the recruitment of the Src-RhoA-phosphoinositide 3-kinase pathway by Vav-2 downstream of G(alpha 13) in pancreatic acini**. *Biochemical and biophysical research communications* 2006, **339**(1):271-276.
2. Daulhac L, Kowalski-Chauvel A, Pradayrol L, Vaysse N, Seva C: **Gastrin stimulates the formation of a p60Src/p125FAK complex upstream of the phosphatidylinositol 3-kinase signaling pathway**. *FEBS letters* 1999, **445**(2–3):251-255.
3. Kowalski-Chauvel A, Pradayrol L, Vaysse N, Seva C: **Gastrin stimulates tyrosine phosphorylation of insulin receptor substrate 1 and its association with Grb2 and the phosphatidylinositol 3-kinase**. *The Journal of biological chemistry* 1996, **271**(42):26356-26361.

4. Daulhac L, Kowalski-Chauvel A, Pradayrol L, Vaysse N, Seva C: **Src-family Tyrosine Kinases in Activation of ERK-1 and p85/p110-phosphatidylinositol 3-Kinase by G/CCKBReceptors**. *Journal of Biological Chemistry* 1999, **274**(29):20657-20663.
5. Ferrand A, Kowalski-Chauvel A, Bertrand C, Pradayrol L, Fourmy D, Dufresne M, Seva C: **Involvement of JAK2 upstream of the PI 3-kinase in cell–cell adhesion regulation by gastrin**. *Experimental Cell Research* 2004, **301**(2):128-138.
6. Todisco A, Ramamoorthy S, Witham T, Pausawasdi N, Srinivasan S, Dickinson CJ, Askari FK, Krametter D: **Molecular mechanisms for the antiapoptotic action of gastrin**. *American journal of physiology Gastrointestinal and liver physiology* 2001, **280**(2):G298-307.
7. Brader S, Eccles SA: **Phosphoinositide 3-kinase signalling pathways in tumor progression, invasion and angiogenesis**. *Tumori* 2004, **90**(1):2-8.
8. Seva C, Kowalski-Chauvel A, Daulhac L, Barthez C, Vaysse N, Pradayrol L: **Wortmannin-Sensitive Activation of p70S6-Kinase and MAP-Kinase by the G Protein-Coupled Receptor, G/CCKB**. *Biochemical and biophysical research communications* 1997, **238**(1):202-206.
9. Subramaniam D, Ramalingam S, May R, Dieckgraefe BK, Berg DE, Pothoulakis C, Houchen CW, Wang TC, Anant S: **Gastrin-Mediated Interleukin-8 and Cyclooxygenase-2 Gene Expression: Differential Transcriptional and Posttranscriptional Mechanisms**. *Gastroenterology* 2008, **134**(4):1070-1082.
10. Minden A, Lin A, Smeal T, Derijard B, Cobb M, Davis R, Karin M: **c-Jun N-terminal phosphorylation correlates with activation of the JNK subgroup but not the ERK subgroup of mitogen-activated protein kinases**. *Mol Cell Biol* 1994, **14**(10):6683-6688.
11. Coronella-Wood J, Terrand J, Sun H, Chen QM: **c-Fos phosphorylation induced by H2O2 prevents proteasomal degradation of c-Fos in cardiomyocytes**. *The Journal of biological chemistry* 2004, **279**(32):33567-33574.
12. Livingstone C, Patel G, Jones N: **Atf-2 Contains a Phosphorylation-Dependent Transcriptional Activation Domain**. *Embo Journal* 1995, **14**(8):1785-1797.
13. Gupta S, Campbell D, Derijard B, Davis RJ: **Transcription Factor Atf2 Regulation by the Jnk Signal-Transduction Pathway**. *Science* 1995, **267**(5196):389-393.
14. He H, Yim M, Liu KH, Cody SC, Shulkes A, Baldwin GS: **Involvement of G proteins of the Rho family in the regulation of Bcl-2-like protein expression and caspase 3 activation by Gastrins**. *Cellular signalling* 2008, **20**(1):83-93.
15. Pritchard DM, Berry D, Przemeck SMC, Campbell F, Edwards SW, Varro A: **Gastrin increases mcl-1 expression in type I gastric carcinoid tumors and a gastric epithelial cell line that expresses the CCK-2 receptor**. *American Journal of Physiology - Gastrointestinal and Liver Physiology* 2008, **295**(4):G798-G805.
16. He H, Baldwin GS: **Rho GTPases and p21-activated kinase in the regulation of proliferation and apoptosis by gastrins**. *The international journal of biochemistry & cell biology* 2008, **40**(10):2018-2022.
17. Michels J, Johnson PWM, Packham G: **Mcl-1**. *The International Journal of Biochemistry & Cell Biology* 2005, **37**(2):267-271.
18. Schürmann A, Mooney AF, Sanders LC, Sells MA, Wang HG, Reed JC, Bokoch GM: **p21-Activated Kinase 1 Phosphorylates the Death Agonist Bad and Protects Cells from Apoptosis**. *Molecular and Cellular Biology* 2000, **20**(2):453-461.
19. Ramamoorthy S, Stepan V, Todisco A: **Intracellular mechanisms mediating the anti-apoptotic action of gastrin**. *Biochemical and biophysical research communications* 2004, **323**(1):44-48.
20. Mishra P, Senthivinayagam S, Rana A, Rana B: **Glycogen Synthase Kinase-3beta regulates Snail and beta-catenin during gastrin-induced migration of gastric cancer cells**. *Journal of molecular signaling* 2010, **5**:9.
21. He H, Shulkes A, Baldwin GS: **PAK1 interacts with beta-catenin and is required for the regulation of the beta-catenin signalling pathway by gastrins**. *Biochimica et biophysica acta* 2008, **1783**(10):1943-1954.

22. Ozcelebi F, Rao RV, Holicky E, Madden BJ, McCormick DJ, Miller LJ: **Phosphorylation of cholecystokinin receptors expressed on Chinese hamster ovary cells. Similarities and differences relative to native pancreatic acinar cell receptors**. *The Journal of biological chemistry* 1996, **271**(7):3750-3755.
23. Rao RV, Roettger BF, Hadac EM, Miller LJ: **Roles of cholecystokinin receptor phosphorylation in agonist-stimulated desensitization of pancreatic acinar cells and receptor-bearing Chinese hamster ovary cholecystokinin receptor cells**. *Mol Pharmacol* 1997, **51**(2):185-192.
24. Pohl M, Silvente-Poirot S, Pisegna JR, Tarasova NI, Wank SA: **Ligand-induced internalization of cholecystokinin receptors. Demonstration of the importance of the carboxyl terminus for ligand-induced internalization of the rat cholecystokinin type B receptor but not the type A receptor**. *The Journal of biological chemistry* 1997, **272**(29):18179-18184.
25. Tarasova NI, Stauber RH, Choi JK, Hudson EA, Czerwinski G, Miller JL, Pavlakis GN, Michejda CJ, Wank SA: **Visualization of G Protein-coupled Receptor Trafficking with the Aid of the Green Fluorescent Protein**. *Journal of Biological Chemistry* 1997, **272**(23):14817-14824.
26. Suzuki M, Raab G, Moses MA, Fernandez CA, Klagsbrun M: **Matrix Metalloproteinase-3 Releases Active Heparin-binding EGF-like Growth Factor by Cleavage at a Specific Juxtamembrane Site**. *Journal of Biological Chemistry* 1997, **272**(50):31730-31737.
27. Miyazaki Y, Shinomura Y, Tsutsui S, Zushi S, Higashimoto Y, Kanayama S, Higashiyama S, Taniguchi N, Matsuzawa Y: **Gastrin induces heparin-binding epidermal growth factor-like growth factor in rat gastric epithelial cells transfected with gastrin receptor**. *Gastroenterology* 1999, **116**(1):78-89.
28. Sinclair NF, Ai W, Raychowdhury R, Bi M, Wang TC, Koh TJ, McLaughlin JT: **Gastrin regulates the heparin-binding epidermal-like growth factor promoter via a PKC/EGFR-dependent mechanism**. *American Journal of Physiology - Gastrointestinal and Liver Physiology* 2004, **286**(6):G992-G999.
29. Sakaguchi K, Okabayashi Y, Kido Y, Kimura S, Matsumura Y, Inushima K, Kasuga M: **Shc phosphotyrosine-binding domain dominantly interacts with epidermal growth factor receptors and mediates Ras activation in intact cells**. *Mol Endocrinol* 1998, **12**(4):536-543.
30. Okutani T, Okabayashi Y, Kido Y, Sugimoto Y, Sakaguchi K, Matuoka K, Takenawa T, Kasuga M: **Grb2/Ash binds directly to tyrosines 1068 and 1086 and indirectly to tyrosine 1148 of activated human epidermal growth factor receptors in intact cells**. *The Journal of biological chemistry* 1994, **269**(49):31310-31314.
31. Seva C, Kowalski-Chauvel A, Blanchet JS, Vaysse N, Pradayrol L: **Gastrin induces tyrosine phosphorylation of Shc proteins and their association with the Grb2/Sos complex**. *FEBS letters* 1996, **378**(1):74-78.
32. Daulhac L, Kowalski-Chauvel A, Pradayrol L, Vaysse N, Seva C: **Ca2+ and protein kinase C-dependent mechanisms involved in gastrin-induced Shc/Grb2 complex formation and P44-mitogen-activated protein kinase activation**. *The Biochemical journal* 1997, **325 (Pt 2)**:383-389.
33. Magnan R, Masri B, Escrieut C, Foucaud M, Cordelier P, Fourmy D: **Regulation of Membrane Cholecystokinin-2 Receptor by Agonists Enables Classification of Partial Agonists as Biased Agonists**. *Journal of Biological Chemistry* 2011, **286**(8):6707-6719.
34. Yu H-G, Schrader H, Otte J-M, Schmidt WE, Schmitz F: **Rapid tyrosine phosphorylation of focal adhesion kinase, paxillin, and p130Cas by gastrin in human colon cancer cells**. *Biochemical Pharmacology* 2004, **67**(1):135-146.
35. Rozengurt E, Walsh JH: **GASTRIN, CCK, SIGNALING, AND CANCER**. *Annual review of physiology* 2001, **63**(1):49-76.
36. Dehez S, Bierkamp C, Kowalski-Chauvel A, Daulhac L, Escrieut C, Susini C, Pradayrol L, Fourmy D, Seva C: **c-Jun NH2-terminal Kinase Pathway in Growth-promoting Effect of the G Protein-coupled Receptor Cholecystokinin B Receptor: A Protein Kinase C/Src-dependent-Mechanism**. *Cell Growth Differ* 2002, **13**(8):375-385.

37. Tapia Ja, Ferris Ha, Jensen RT, García LJ: **Cholecystokinin activates PYK2/CAKbeta by a phospholipase C-dependent mechanism and its association with the mitogen-activated protein kinase signaling pathway in pancreatic acinar cells.** *The Journal of biological chemistry* 1999, **274**:31261-31271.
38. Pace A, García-Marin LJ, Tapia Ja, Bragado MJ, Jensen RT: **Phosphospecific site tyrosine phosphorylation of p125FAK and proline-rich kinase 2 is differentially regulated by cholecystokinin receptor type A activation in pancreatic acini.** *The Journal of biological chemistry* 2003, **278**:19008-19016.
39. Mitra SK, Hanson Da, Schlaepfer DD: **Focal adhesion kinase: in command and control of cell motility.** *Nature reviews Molecular cell biology* 2005, **6**:56-68.
40. Sancho V, Berna MJ, Thill M, Jensen RT: **PKCθ activation in pancreatic acinar cells by gastrointestinal hormones/neurotransmitters and growth factors is needed for stimulation of numerous important cellular signaling cascades**. *Biochimica et Biophysica Acta (BBA) - Molecular Cell Research* 2011, **1813**(12):2145-2156.
41. Lipinski CA, Loftus JC: **Targeting Pyk2 for therapeutic intervention.** *Expert opinion on therapeutic targets* 2010, **14**:95-108.
42. Blaukat A, Ivankovic-Dikic I, Grönroos E, Dolfi F, Tokiwa G, Vuori K, Dikic I: **Adaptor proteins Grb2 and Crk couple Pyk2 with activation of specific mitogen-activated protein kinase cascades.** *The Journal of biological chemistry* 1999, **274**:14893-14901.
43. Andreolotti AG, Bragado MJ, Tapia Ja, Jensen RT, Garcia-Marin LJ: **Adapter protein CRKII signaling is involved in the rat pancreatic acini response to reactive oxygen species.** *Journal of cellular biochemistry* 2006, **97**:359-367.
44. Seufferlein T, Withers D, Broad S, Herget T, Walsh J, Rozengurt E: **The human CCKB/gastrin receptor transfected into rat1 fibroblasts mediates activation of MAP kinase, p74raf-1 kinase, and mitogenesis**. *Cell Growth Differ* 1995, **6**(4):383-393.
45. Hocker M: **Molecular mechanisms of gastrin-dependent gene regulation**. *Annals of the New York Academy of Sciences* 2004, **1014**:97-109.
46. Anjum R, Blenis J: **The RSK family of kinases: emerging roles in cellular signalling**. *Nat Rev Mol Cell Biol* 2008, **9**(10):747-758.
47. Hocker M, Raychowdhury R, Plath T, Wu H, O'Connor DT, Wiedenmann B, Rosewicz S, Wang TC: **Sp1 and CREB mediate gastrin-dependent regulation of chromogranin A promoter activity in gastric carcinoma cells**. *The Journal of biological chemistry* 1998, **273**(51):34000-34007.
48. Guo Y-S, Cheng J-Z, Jin G-F, Gutkind JS, Hellmich MR, Townsend CM: **Gastrin Stimulates Cyclooxygenase-2 Expression in Intestinal Epithelial Cells through Multiple Signaling Pathways**. *Journal of Biological Chemistry* 2002, **277**(50):48755-48763.
49. Dehez S, Daulhac L, Kowalski-Chauvel A, Fourmy D, Pradayrol L, Seva C: **Gastrin-induced DNA synthesis requires p38-MAPK activation via PKC/Ca2+ and Src-dependent mechanisms**. *FEBS letters* 2001, **496**(1):25-30.
50. Satoh A, Gukovskaya AS, Nieto JM, Cheng JH, Gukovsky I, Reeve JR, Shimosegawa T, Pandol SJ: **PKC-delta and -epsilon regulate NF-kappa B activation induced by cholecystokinin and TNF-alpha in pancreatic acinar cells**. *Am J Physiol-Gastr L* 2004, **287**(3):G582-G591.
51. Yuan JZ, Lugea A, Zheng L, Gukovsky I, Edderkaoui M, Rozengurt E, Pandol SJ: **Protein kinase D1 mediates NF-kappa B activation induced by cholecystokinin and cholinergic signaling in pancreatic acinar cells**. *Am J Physiol-Gastr L* 2008, **295**(6):G1190-G1201.
52. Koh YH, Tamizhselvi R, Bhatia M: **Extracellular Signal-Regulated Kinase 1/2 and c-Jun NH2-Terminal Kinase, through Nuclear Factor-kappa B and Activator Protein-1, Contribute to Caerulein-Induced Expression of Substance P and Neurokinin-1 Receptors in Pancreatic Acinar Cells**. *J Pharmacol Exp Ther* 2010, **332**(3):940-948.
53. Varro A, Noble P-JM, Pritchard DM, Kennedy S, Hart CA, Dimaline R, Dockray GJ: **Helicobacter pylori Induces Plasminogen Activator Inhibitor 2 in Gastric Epithelial Cells through Nuclear Factor-κB and RhoA**. *Cancer research* 2004, **64**(5):1695-1702.

54. Moustafa A, Sakamoto KQ, Habara Y: **A fundamental role for NO-PLC signaling pathway in mediating intracellular Ca2+ oscillation in pancreatic acini.** *Nitric oxide : biology and chemistry / official journal of the Nitric Oxide Society* 2011, **24**:139-150.
55. Cordelier P, Estève JP, Bousquet C, Delesque N, O'Carroll aM, Schally aV, Vaysse N, Susini C, Buscail L: **Characterization of the antiproliferative signal mediated by the somatostatin receptor subtype sst5.** *Proceedings of the National Academy of Sciences of the United States of America* 1997, **94**:9343-9348.
56. Cordelier P, Estève JP, Rivard N, Marletta M, Vaysse N, Susini C, Buscail L: **The activation of neuronal NO synthase is mediated by G-protein betagamma subunit and the tyrosine phosphatase SHP-2.** *The FASEB journal : official publication of the Federation of American Societies for Experimental Biology* 1999, **13**:2037-2050.
57. Arena S, Pattarozzi A, Corsaro A, Schettini G, Florio T: **Somatostatin receptor subtype-dependent regulation of nitric oxide release: involvement of different intracellular pathways.** *Molecular endocrinology (Baltimore, Md)* 2005, **19**:255-267.
58. Sternfeld L, Krause E, Guse AH, Schulz I: **Hormonal control of ADP-ribosyl cyclase activity in pancreatic acinar cells from rats.** *The Journal of biological chemistry* 2003, **278**:33629-33636.
59. Lee HC: **Cyclic ADP-ribose and NAADP: fraternal twin messengers for calcium signaling.** *Science China Life sciences* 2011, **54**:699-711.
60. Thorn P, Gerasimenko O, Petersen OH: **Cyclic ADP-ribose regulation of ryanodine receptors involved in agonist evoked cytosolic Ca2+ oscillations in pancreatic acinar cells.** *The EMBO journal* 1994, **13**:2038-2043.
61. Fitzsimmons TJ, Gukovsky I, McRoberts Ja, Rodriguez E, Lai Fa, Pandol SJ: **Multiple isoforms of the ryanodine receptor are expressed in rat pancreatic acinar cells.** *The Biochemical journal* 2000, **351**:265-271.
62. Cancela JM, Mogami H, Tepikin aV, Petersen OH: **Intracellular glucose switches between cyclic ADP-ribose and inositol trisphosphate triggering of cytosolic Ca2+ spiking.** *Current biology : CB* 1998, **8**:865-868.
63. Sjodin L, Gardner JD: **Effect of Cholecystokinin Variant (Cck39) on Dispersed Acinar Cells from Guinea-Pig Pancreas**. *Gastroenterology* 1977, **73**(5):1015-1018.
64. Meinkoth JL, Alberts AS, Went W, Fantozzi D, Taylor SS, Hagiwara M, Montminy M, Feramisco JR: **Signal-Transduction through the Camp-Dependent Protein-Kinase**. *Mol Cell Biochem* 1993, **128**:179-186.
65. Kim C, Cheng CY, Saldanha SA, Taylor SS: **PKA-I holoenzyme structure reveals a mechanism for cAMP-dependent activation**. *Cell* 2007, **130**(6):1032-1043.
66. Naviglio S, Caraglia M, Abbruzzese A, Chiosi E, Di Gesto D, Marra M, Romano M, Sorrentino A, Sorvillo L, Spina A *et al*: **Protein kinase A as a biological target in cancer therapy**. *Expert Opin Ther Tar* 2009, **13**(1):83-92.
67. Dabrowski A, Groblewski GE, Schafer C, Guan KL, Williams JA: **Cholecystokinin and EGF activate a MAPK cascade by different mechanisms in rat pancreatic acinar cells**. *Am J Physiol-Cell Ph* 1997, **273**(5):C1472-C1479.
68. Morrison DK, Cutler Jr RE: **The complexity of Raf-1 regulation**. *Current Opinion in Cell Biology* 1997, **9**(2):174-179.
69. Zimmermann S, Moelling K: **Phosphorylation and Regulation of Raf by Akt (Protein Kinase B)**. *Science* 1999, **286**(5445):1741-1744.
70. Booden MA, Siderovski DP, Der CJ: **Leukemia-associated Rho guanine nucleotide exchange factor promotes G alpha q-coupled activation of RhoA**. *Mol Cell Biol* 2002, **22**(12):4053-4061.
71. Dabrowski A, VanderKuur JA, CarterSu C, Williams JA: **Cholecystokinin stimulates formation of Shc-Grb2 complex in rat pancreatic acinar cells through a protein kinase C-dependent mechanism**. *Journal of Biological Chemistry* 1996, **271**(43):27125-27129.

72. Daulhac L, Kowalski-Chauvel A, Pradayrol L, Vaysse N, Seva C: **Src-family tyrosine kinases in activation of ERK-1 and p85/p110-phosphatidylinositol 3-kinase by G/CCKB receptors**. *The Journal of biological chemistry* 1999, **274**(29):20657-20663.
73. Blaukat A, Ivankovic-Dikic I, Gronroos E, Dolfi F, Tokiwa G, Vuori K, Dikic I: **Adaptor proteins Grb2 and Crk couple Pyk2 with activation of specific mitogen-activated protein kinase cascades**. *Journal of Biological Chemistry* 1999, **274**(21):14893-14901.
74. Tapia JA, Ferris HA, Jensen RT, Garcia LJ: **Cholecystokinin activates PYK2/CAK beta by a phospholipase C-dependent mechanism and its association with the mitogen-activated protein kinase signaling pathway in pancreatic acinar cells**. *Journal of Biological Chemistry* 1999, **274**(44):31261-31271.
